# Hepatic phosphate uptake and subsequent nerve-mediated phosphaturia are crucial for phosphate homeostasis following portal vein passage of phosphate in rats

**DOI:** 10.1038/s41598-023-32856-2

**Published:** 2023-04-08

**Authors:** Seiichi Yasuda, Kazunori Inoue, Isao Matsui, Ayumi Matsumoto, Yusuke Katsuma, Hiroki Okushima, Atsuhiro Imai, Yusuke Sakaguchi, Jun-ya Kaimori, Ryohei Yamamoto, Masayuki Mizui, Yoshitaka Isaka

**Affiliations:** 1 Department of Nephrology, Osaka University Graduate School of Medicine, 2-2 Yamada-oka, Suita, Osaka, 565-0871 Japan; 2 Transdimensional Life Imaging Division, Institute for Open and Transdisciplinary Research Initiatives, Osaka University, 2-1 Yamada-oka, Suita, Osaka, 565-0871 Japan; 3 Department of Inter-Organ Communication Research in Kidney Disease, Osaka University Graduate School of Medicine, 2-2 Yamada-oka, Suita, Osaka, 565-0871 Japan; 4 Health Promotion and Regulation, Department of Health Promotion Medicine, Osaka University Graduate School of Medicine, 1-17 Machikaneyamacho, Toyonaka, Osaka, 560-0043 Japan; 5 Health and Counseling Center, Osaka University, 1-17 Machikaneyamacho, Toyonaka, Osaka, 560-0043 Japan

**Keywords:** Calcium and vitamin D, Homeostasis

## Abstract

Fibroblast growth factor 23, parathyroid hormone, and 1,25-dihydroxyvitamin D are critical in phosphate homeostasis. Despite these factors’ importance, regulators of phosphaturia in the acute postprandial phase remain largely unknown. This study investigated the mechanism of acute phosphate regulation in the postprandial phase in rats. Duodenal administration of radiolabeled phosphate (^32^P) showed that ^32^P levels in the inferior vena cava (IVC) blood were lower than those in the portal vein (PV) blood. Serum phosphate concentration transiently increased 5 min after phosphate solution administration through IVC, while it was maintained after the administration through PV. Phosphate administration through both IVC and PV resulted in increased fractional excretion of phosphate (FEPi) at 10 min without elevation of the known circulating factors, but urinary phosphate excretion during the period was 8% of the dose. Experiments using ^32^P or partial hepatectomy showed that the liver was one of the phosphate reservoirs. The elevation of FEPi and suppression of sodium-phosphate cotransporter 2a in the kidney at 10 min was attenuated in rats with SCH23390, hepatic denervation, or renal denervation, thus indicating that the liver communicated with the kidney via the nervous system to promote phosphaturia. These results revealed previously unknown mechanisms for serum phosphate maintenance.

## Introduction

Phosphate is essential for various biological processes, and its deficiency and excess have been shown to cause various disorders^1–8^. Therefore, understanding the homeostatic mechanisms associated with phosphate is very important.

The kidney plays an essential role in maintaining phosphate homeostasis. High dietary phosphate for several days in healthy humans has been shown to increase intact fibroblast growth factor 23 (i-FGF23) and intact parathyroid hormone (i-PTH) and suppress 1,25-dihydroxyvitamin D (1,25VD) levels, leading to the elevation of urinary phosphate excretion^9–11^. Despite the importance of these circulating factors on a time axis of several days, regulators of phosphate in the acute postprandial phase remain largely unknown. For example, Isakova et al. demonstrated that healthy human volunteers increased fractional excretion of phosphate (FEPi) within 30 min after a meal (phosphorus 500 mg) without elevating serum phosphate, calcium, and i-FGF23 levels, while i-PTH levels rather decreased^12^. In animal experiments, Berndt et al. showed that duodenal phosphate administration (1.3 mmol/rat) increased duodenum-derived phosphaturic factor and thereby increased FEPi within 10 min without affecting plasma levels of FGF23 and PTH^13^. However, Thomas et al. and Scanni et al. found no evidence for intestinal-specific phosphaturic factor in rats and humans, respectively^14,15^. Thomas et al. examined the response of rats, pretreated with a low phosphate diet followed by overnight fasting, to intravenous or intestinal phosphate bolus (0.5 mmol/rat)^14^. They concluded that phosphate bolus causes rapid phosphaturia through mechanisms requiring PTH. Scanni et al. demonstrated that phosphaturic response occurs after the elevation of plasma phosphate and PTH in either group of healthy humans underwent intravenous (1.15 and 2.30 mmol/kg/day) or duodenal (1.53 mmol/kg/day) phosphate loading^15^. Taken together, these results may indicate that the PTH-mediated mechanism plays an important role in acutely maintaining phosphate homeostasis under high phosphate loads. Still, acute regulators under moderate phosphate loading conditions remain unknown, as shown in Isakova's study^12^.

In this study, we investigated the mechanism of acute phosphate regulation under moderate phosphate loading in rats. Rats received phosphate solution under conditions that did not alter the concentrations of circulating phosphate, calcium, 1,25VD, i-PTH, and i-FGF23. We performed our experiments on rats fed a regular diet without fasting. Since the diet is the primary source of phosphate loading to the body, we thought the fed condition would be preferable for studying phosphate kinetics. Our results provide novel insights into the maintenance of phosphate homeostasis.

## Results

### Phosphate administration through the duodenum results in phosphate uptake in the liver and bone

**Figure 1:**
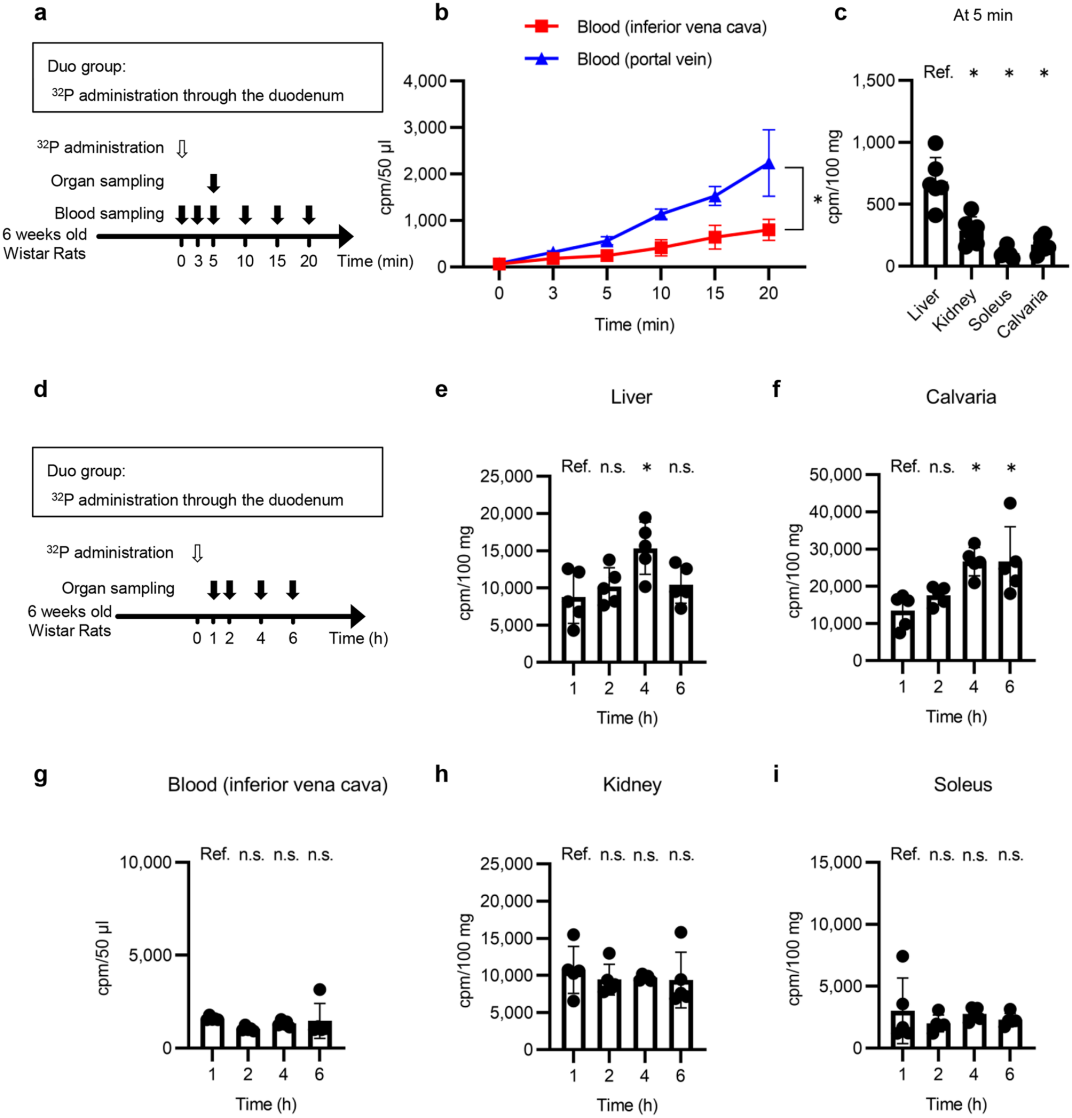
Phosphate kinetics in rats after phosphate administration through the duodenum. (**a**) Experimental design of (**b**) and (**c**). The effects of duodenal administration of phosphate on normal rats (Duo group) were analyzed. A Na_2_HPO_4_/NaH_2_PO_4_ solution (phosphate 80 mM, sodium 154 mM, pH 7.4, 1.0 ml/animal) containing trace amounts of radiolabeled phosphate (^32^P, 3700 Bq/ml) was administered through the duodenum at time 0. Blood samples were collected from the inferior vena cava and portal vein at the indicated time points. Organ samples were collected at 5 min. (**b**) ^32^P levels (counts per minute (cpm)) in the blood of the inferior vena cava and portal vein (n = 4 rats per group). (**c**) ^32^P levels in the liver, kidney, soleus, and calvaria at 5 min (n = 6 rats per group). (**d**) Experimental design of (**e**) to (**i**). The radiolabeled sodium phosphate solution was administered in the same manner as in (**a**). Levels of ^32^P in the (**e**) liver, (**f**) calvaria, (**g**) blood from the inferior vena cava, (**h**) kidney, and (**i**) soleus were measured at the indicated time points (n = 5 rats per group). All results are presented as the means ± SDs, n.s.: not significant, Ref.: reference. *P < 0.05 based on the repeated measures of ANOVA in (**b**) and on Dunnett’s test in (**c**) and (**e**) to (**i**).

To analyze phosphate dynamics following intestinal absorption, a sodium phosphate (80 μmol/animal) solution containing ^32^P was administered through the duodenum to normal rats (Duo group) (Fig. 1a). The ^32^P level in the inferior vena cava blood was lower than in portal vein blood (Fig. 1b). We measured ^32^P levels in several organs at 5 min and found that the liver contained the highest level of ^32^P (Fig. 1c). The levels of ^32^P in the liver peaked at 4 h, while those in the calvaria gradually increased during the experimental period (Fig. 1d–f). The levels of ^32^P in the inferior vena cava blood, kidney, and soleus did not change during 1–6 h (Fig. 1g–i).

### Phosphate administration through the portal vein maintains serum phosphate concentration at 5 min in a manner independent of urinary phosphate excretion

**Figure 2:**
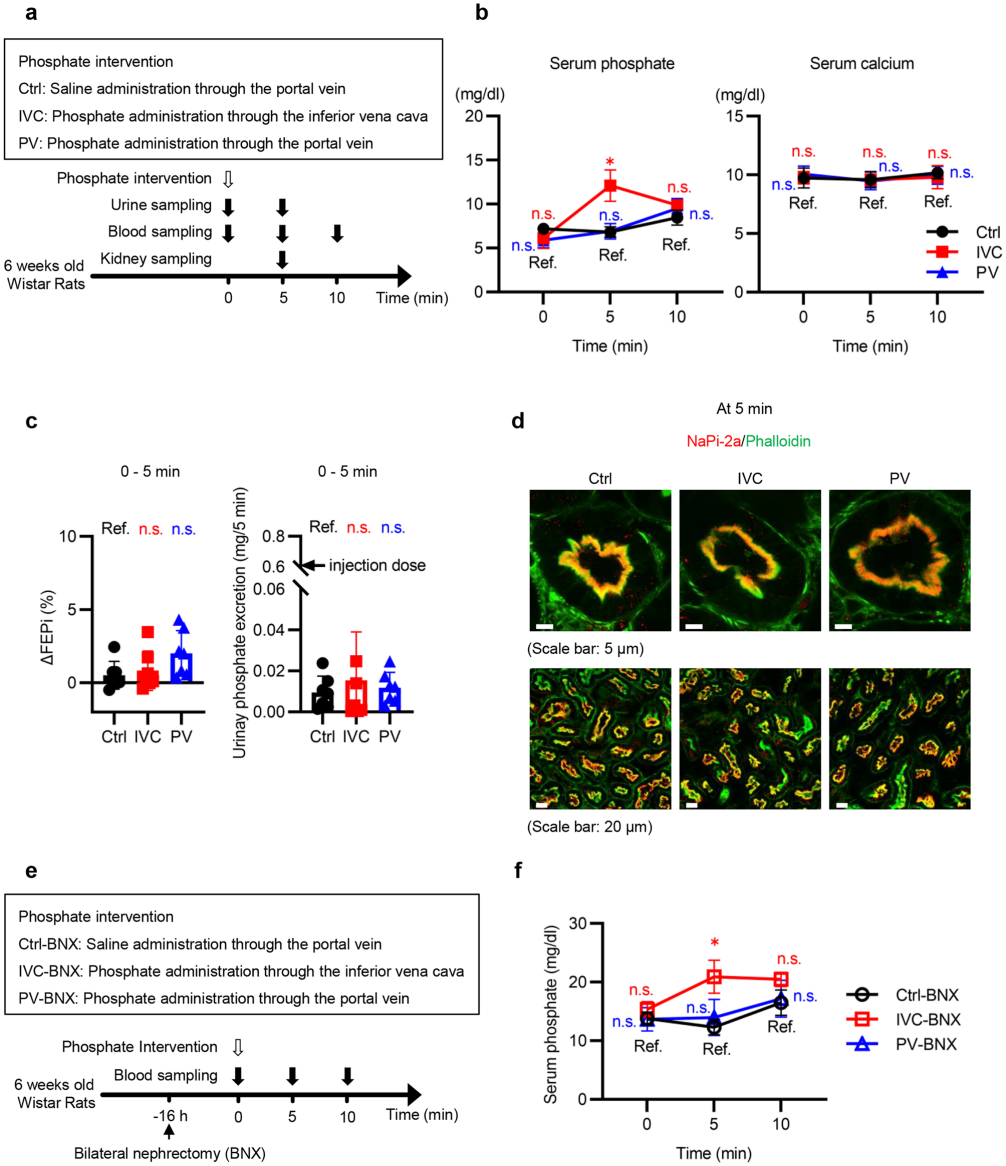
Phosphate administration through the portal vein, not the inferior vena cava, maintained serum phosphate levels at 5 min in a phosphaturia-independent manner. (**a**) Experimental design of (**b**) to (**d**). Effects of phosphate administration through the portal vein or inferior vena cava (hereafter referred to as phosphate intervention) were analyzed. The normal rats were randomly divided into three groups, Ctrl, IVC, and PV. Rats in the IVC group were administered Na_2_HPO_4_/NaH_2_PO_4_ solution (20 mM phosphate, 154 mM sodium, pH 7.4, 1.0 ml/animal) through the inferior vena cava at time 0. Rats in the PV group were administered the same phosphate solution through the portal vein at time 0. Rats in the Ctrl group were administered normal saline (sodium 154 mM, 1.0 ml/animal) through the portal vein. (**b**) Serum phosphate and calcium levels at the indicated times (n = 5 rats per group). Serum samples were collected from the inferior vena cava. (**c**) Changes in fractional excretion of phosphate (ΔFEPi) between 0 and 5 min, and urinary phosphate excretion during the 5-min period (n = 7 rats per group). (**d**) Representative immunofluorescence images of renal proximal tubular epithelial cells (PTECs) at 5 min. Kidney sections were stained with phalloidin (green) and sodium-phosphate cotransporter 2a (NaPi-2a) (red). The upper and the lower images in each group are the images with higher and lower magnifications, respectively (scale bars: 5 µm or 20 µm). (**e**) Experimental design of (**f**). Bilaterally nephrectomized (BNX) rats were divided into Ctrl-BNX, IVC-BNX, and PV-BNX groups. Sixteen hours after the BNX operation, rats in the Ctrl-BNX, IVC-BNX, and PV-BNX groups similarly received phosphate interventions to the rats in the Ctrl, IVC, and PV groups, respectively. (**f**) Serum phosphate concentrations in the BNX rats at the indicated times (n = 5 rats per group). All results are presented as the means ± SDs, ns: not significant, Ref: reference. *P < 0.05 based on Dunnett’s test in (**c**). Repeated measures ANOVA is useful for analyzing mean score changes over three or more time points. However, Dunnett’s test was applied in (**b**) and (**f**) to detect temporal changes in serum phosphate levels, not mean changes over time. Considering type 1 error by multiple measurements (0, 5, and 10 min), Bonferroni correction was also applied. *P < 0.025 based on Dunnett’s test with Bonferroni correction in (**b**) and (**f**).

We sought to determine whether phosphate absorption from the intestine, increased serum phosphate concentration, or decreased serum calcium concentration is an essential trigger for maintaining phosphate homeostasis. For this purpose, a phosphate solution was administered to normal rats through the portal vein, which bypasses intestinal absorption, at a dose that did not decrease serum calcium concentrations. We decided to administer 20 μmol/animal phosphate because 100 μmol/animal marginally and 500 μmol/animal significantly lowered serum calcium (Supplementary Fig. S1 online). Since rats weighing 150–170 g were used in this study, the amount corresponds to a phosphorus dose of 219–248 mg for a human weighing 60 kg. The normal rats were randomly divided into three groups, Ctrl, IVC, and PV. Rats in the Ctrl group were administered normal saline through the portal vein, while rats in the IVC and PV groups received 20 µmol/animal of phosphate through the inferior vena cava and portal vein, respectively (Fig. 2a). In all of the following experiments, except for radiolabeled assays, rats in groups whose group name contains Ctrl, IVC, and PV were subjected to phosphate interventions similarly to the rats in the Ctrl, IVC, and PV groups, respectively. The phosphate intervention increased serum phosphate concentration at 5 min in the IVC group but not in the PV group (Fig. 2b). Serum calcium levels were not affected (Fig. 2b). Serum phosphate levels gradually increased over time, possibly due to anesthesia, even in the Ctrl group (Fig. 2b). In addition to differences in FEPi between 0 and 5 min in each group, we evaluated differences in the changes in fractional excretion of phosphate (FEPi) (ΔFEPi) between 0 and 5 min among the three groups. Although FEPi in the PV group at 5 min was higher than that at 0 min, ΔFEPi was not different among the three groups (Fig. 2c and Supplementary Fig. S2a online). The amount of urinary phosphate excretion was approximately 3% of the injected dose (Fig. 2c). The localization of NaPi-2a in the kidney at 5 min was similar among the three groups (Fig. 2d). The phosphate interventions did not affect creatinine clearance (Ccr) (Supplementary Fig. S2b online). We performed experiments similar to those in Fig. 2a using bilateral nephrectomy (BNX) rats to exclude the effect of urinary phosphate excretion (Fig. 2e). Serum phosphate levels at 5 and 10 min were elevated in the IVC-BNX group but not in the PV-BNX group (Fig. 2f).

### Hepatic uptake of phosphate plays an essential role in the maintenance of serum phosphate levels in the PV group at 5 min

**Figure 3:**
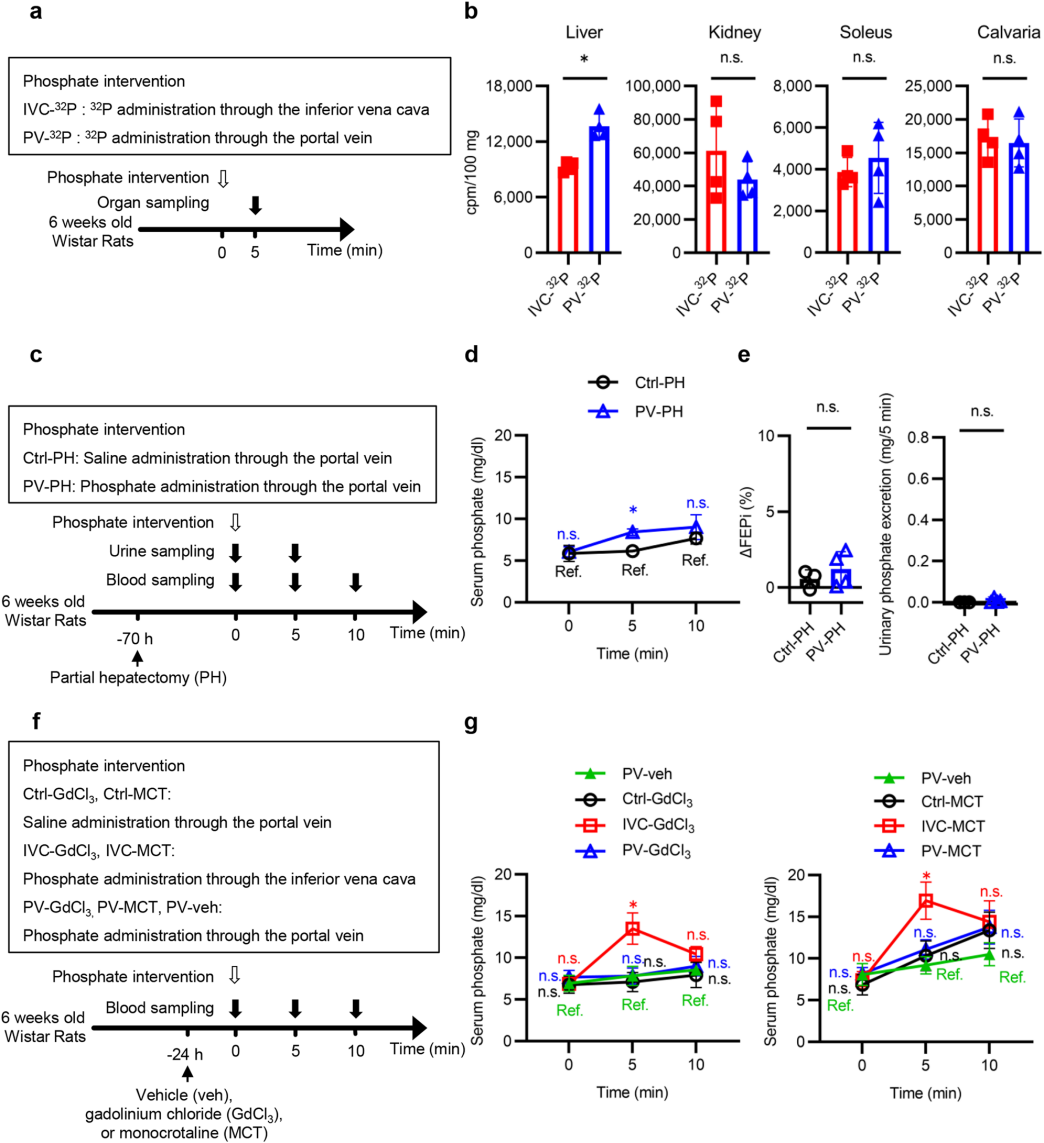
Hepatic phosphate uptake is essential in maintaining serum phosphate levels in the PV group at 5 min. (**a**) Experimental design of (**b**). The normal rats were randomly divided into two groups, IVC-^32^P and PV-^32^P. The rats were administered a radiolabeled Na_2_HPO_4_/NaH_2_PO_4_ solution (20 mM phosphate, 154 mM sodium, pH 7.4, 3700 Bq/ml ^32^P, 1.0 ml/animal) through either the inferior vena cava or portal vein at 0 min. (**b**) ^32^P levels in the liver, kidney, soleus, and calvaria at 5 min (n = 4 rats per group). (**c**) Experimental design of (**d**) and (**e**). Partially hepatectomized (PH) rats were randomly divided into Ctrl-PH and PV-PH groups. Seventy hours after the PH operation, rats in the Ctrl-PH and PV-PH groups received phosphate interventions as in the Ctrl and PV groups. (**d**) Serum phosphate concentration at the indicated times (n = 4 rats per group). Serum samples were collected from the inferior vena cava. (**e**) ΔFEPi between 0 and 5 min and urinary phosphate excretion during the 5-min period (n = 4 rats per group). (**f**) Experimental design of (**g**). Gadolinium chloride (GdCl_3_) or monocrotaline (MCT) pretreated rats were further divided into each of the three groups according to the phosphate interventions. Rats in the PV-veh group were pretreated with normal saline. Twenty-four hours after these pretreatments, rats in groups with Ctrl in the group name, rats in groups with IVC in the group name, and rats in groups with PV in the group name were subjected to phosphate interventions similarly to the rats in the Ctrl, IVC, and PV groups, respectively. (**g**) Serum phosphate concentration in GdCl_3_−, MCT-, or vehicle-pretreated rats (n = 4 rats per group). All results are presented as the means ± SDs, n.s.: not significant, Ref: reference. *P < 0.05 based on the two-tailed Student’s *t-*test in (**b**) and (**e**). *P < 0.025 based on the two-tailed Student’s *t-*test in (**d**) or Dunnett’s test in (**g**) with Bonferroni correction.

We examined whether the liver contributed to the maintenance of serum phosphate in the PV group at 5 min. The normal rats were randomly divided into two groups based on whether they were administered ^32^P-containing phosphate solution (20 µmol) through the inferior vena cava (IVC-^32^P group) or portal vein (PV-^32^P group) (Fig. 3a). The liver in the PV-^32^P group took up a higher level of ^32^P than that in the IVC-^32^P group at 5 min (Fig. 3b). The levels of ^32^P in the kidney, soleus, and calvaria were not different between the two groups. To clarify the role of the liver in maintaining serum phosphate levels, we administered phosphate to partially hepatectomized (PH) rats (Fig. 3c). A previous report has shown that hepatectomy-induced hyperphosphaturia returns to a steady state at 96 h.^16^ Another previous report showed that three days after the 70% hepatectomy, liver regeneration goes to a level equivalent to 20–30% resection^17^. Considering these points, we removed approximately 70% of the liver volume 70 h prior to the phosphate interventions (Fig. 3c). Under our experimental conditions, livers that underwent PH surgery recovered to the equivalent of 14% resection at time 0 (Supplementary Fig. S3a online). Serum phosphate levels in the PV-PH group were higher than those in the Ctrl-PH group at 5 min (Fig. 3d). The difference between the PV-PH and Ctrl-PH groups at 5 min was significant but small because the liver regeneration progressed during the postoperative period. FEPi in each group was not different between 0 and 5 min (Supplementary Fig. S3b online). ΔFEPi between 0 and 5 min was at comparable levels between the two groups, and urinary phosphate excretion during the period was less than the administered phosphate dose (Fig. 3e). Ccr was not different between the Ctrl-PH and the PV-PH groups (Supplementary Fig. S3c online).

Among liver cell types, Kupffer and sinusoidal endothelial cells have been shown to uptake calciprotein particles (CPPs)^18^. We examined whether these cell types contributed to hepatic phosphate uptake. Experiments similar to those in Fig. 2a were carried out in rats whose Kupffer or sinusoidal endothelial cell function was impaired by gadolinium chloride (GdCl_3_) or monocrotaline (MCT), respectively (Fig. 3f). The development of the cell-type-specific dysfunction was confirmed in Supplementary Fig. S3d–f online. We also confirmed that the pretreatments did not affect serum creatinine levels (Supplementary Fig. S3g,h online). Rats were divided into seven groups as shown in Fig. 3f. Serum phosphate levels at 5 min in the PV-GdCl_3_ group were similar to those in the PV-veh and Ctrl-GdCl_3_ groups. The levels in the PV-MCT group were similar to those in the Ctrl-MCT group (Fig. 3g). Serum phosphate levels tended to increase even in the Ctrl-MCT group, which was not administered phosphate, compared with the PV-veh group at 10 min, possibly because the impairment of endothelial cells enhanced the effect of anesthesia on the elevation of serum phosphate concentration in MCT-treated rats.

### FEPi and phosphaturia increased in both the PV and IVC groups in a dopamine D1-like receptor-dependent manner within 10 min

**Figure 4:**
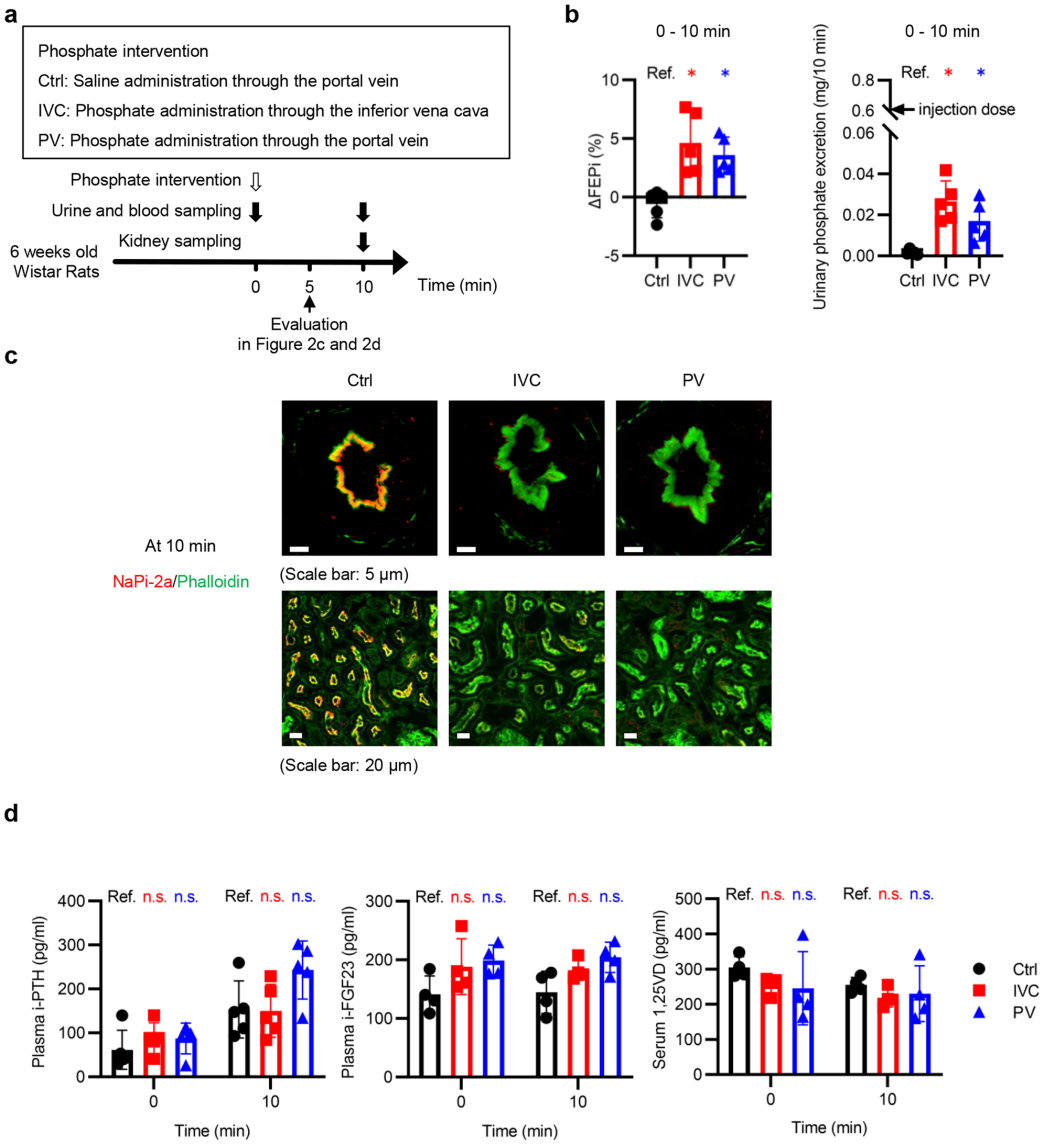
Within 10 min after the phosphate intervention, FEPi and phosphaturia increased in both the PV and CV groups. (**a**) Experimental design of (**b**) to (**d**). The normal rats received phosphate interventions in the same manner as shown in Fig. 2a. The rats at 5 min after the phosphate interventions were evaluated in Fig. 2c,d, while rats at 10 min were evaluated in Fig. 4. (**b**) ΔFEPi between 0 and 10 min and urinary phosphate excretion during the 10-min period (n = 5 rats per group). (**c**) Representative immunofluorescence images of renal PTECs stained with phalloidin (green) and NaPi-2a (red) at 10 min. The upper and the lower images in each group are the immunofluorescence images with higher and lower magnifications, respectively (scale bars: 5 µm or 20 µm). (**d**) Plasma intact-parathyroid hormone (i-PTH), intact-fibroblast growth factor 23 (i-FGF23), and serum 1,25-dihydroxyvitamin D (1,25VD) concentrations at 0 and 10 min (n = 4–5 rats per group). Plasma and serum samples were collected from the inferior vena cava. The results are presented as the means ± SDs, n.s.: not significant, Ref.: reference. *P < 0.05 based on Dunnett’s test in (**b**) and (**d**).

Although the liver was the major contributor to the maintenance of serum phosphate at 5 min in the PV group, it is essential to increase phosphaturia following hepatic uptake to maintain phosphate homeostasis. Therefore, we analyzed urine samples collected during the first 10 min (Fig. 4a). The phosphate intervention did not affect Ccr (Supplementary Fig. S4a online). FEPi in each group was increased in the IVC and the PV groups, but not in the Ctrl group (Supplementary Fig. S4b online). Compared with the Ctrl group, the IVC and PV groups showed greater ΔFEPi between 0 and 10 min and higher urinary phosphate excretion (Fig. 4b). NaPi-2a expression was reduced in both groups at 10 min (Fig. 4c). However, the levels of the three circulating phosphate regulators were not different among the three groups (Fig. 4d). Compared to i-PTH levels at 0 min, i-PTH at 10 min marginally increased (*P* = 0.0678 by the two-tailed Student’s *t-*test) even in the Ctrl group. Therefore, a similar experiment to that shown in Fig. 4a was performed in rats with thyroparathyroidectomy (TPTX) (Supplementary Fig. S4c online). After confirming successful TPTX, the rats were randomly divided into Ctrl-TPTX, IVC-TPTX, and PV-TPTX groups (Supplementary Fig. S4d,e online). Experimental results similar to the Ctrl, IVC, and PV groups were obtained in the Ctrl-TPTX, IVC-TPTX, and PV-TPTX groups (Supplementary Figure S4f–i online).

**Figure 5:**
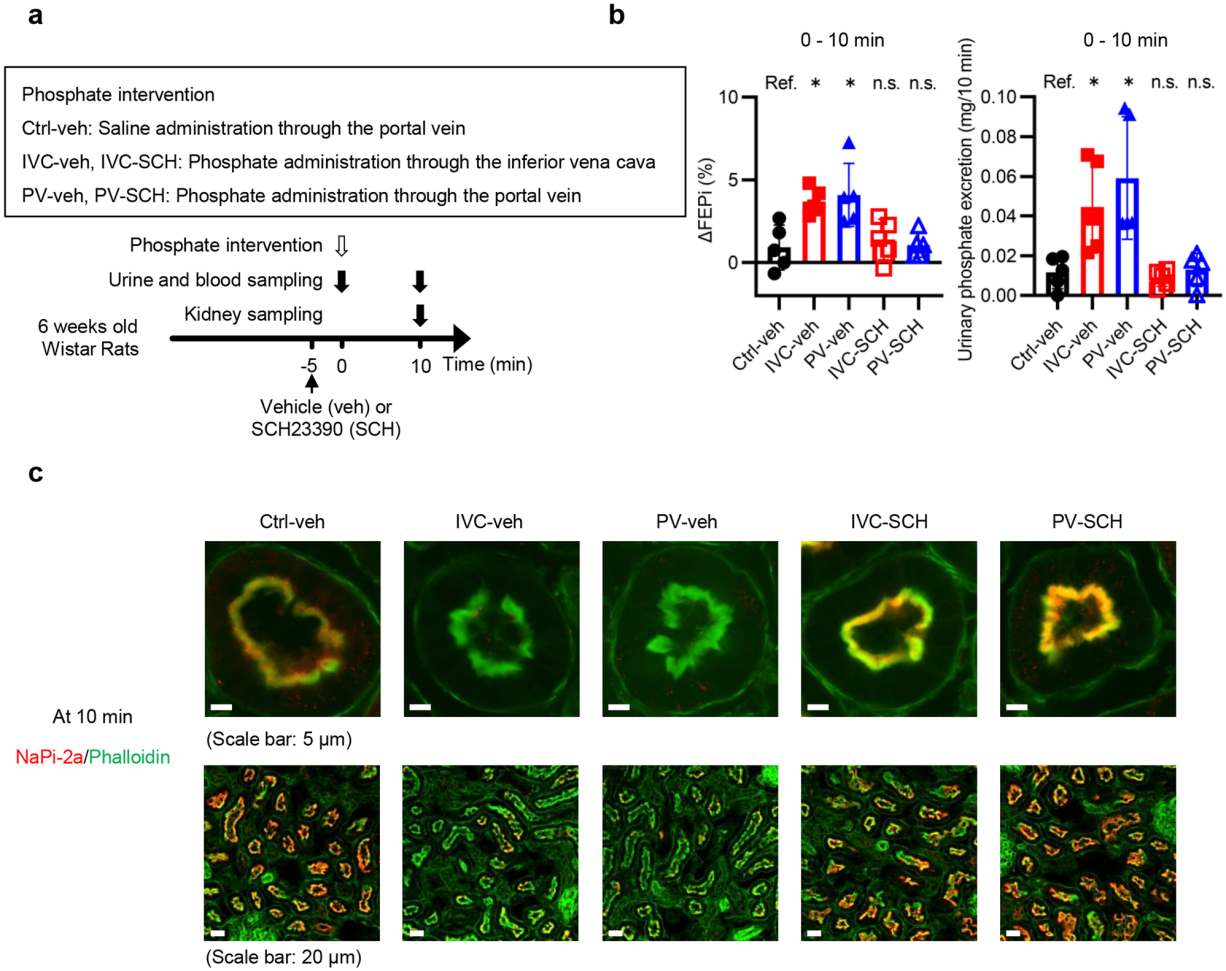
Rats in the IVC and PV groups increased FEPi and phosphaturia within the first 10 min in a dopamine D1-like receptor-dependent manner. (**a**) Experimental design for (**b**) and (**c**). Rats pretreated with SCH23390, a D1-like dopamine receptor antagonist, were randomly divided into two groups, IVC-SCH and PV-SCH. Vehicle-pretreated (normal saline) rats were randomly divided into three groups, Ctrl-veh, IVC-veh, and PV-veh. Five minutes after these pretreatments, rats in groups with Ctrl in the group name, rats in groups with IVC in the group name, and rats in groups with PV in the group name were subjected to phosphate interventions similarly to the rats in the Ctrl, IVC, and PV groups, respectively. (**b**) ΔFEPi between 0 and 10 min and urinary phosphate excretion during the 10-min period (n = 5 rats per group). (**c**) Representative immunofluorescence images of renal PTECs stained with phalloidin (green) and NaPi-2a (red) at 10 min. The upper and the lower images in each group are the immunofluorescence images with higher and lower magnification, respectively (scale bars: 5 µm or 20 µm). The results are presented as the means ± SDs, n.s.: not significant, Ref.: reference. *P < 0.05 based on Dunnett’s test in (**b**).

We analyzed nicotine amide dinucleotide (NAD) levels and mRNA expression of nicotinamide phosphoribosyltransferase (*Nampt*) in the kidneys of the rats shown in Fig. 4a because NAD and *Nampt* have been reported to regulate phosphaturia^16^. There was no difference in NAD and *Nampt* mRNA levels among the three groups (Supplementary Fig. S5 online). We further examined the effects of SCH23390, a selective dopamine D1-like receptor antagonist, because activation of a dopamine D1-like receptor has been reported to be another factor that internalizes NaPi-2a^19^. Rats pretreated with vehicle or SCH23390 received the phosphate interventions (Fig. 5a). In the phosphate-loaded groups either with or without SCH23390, FEPi at 10 min was higher than that at 0 min (Supplementary Fig. S6a online), but the magnitude of the increase in FEPi and the amount of urinary phosphate excretion were significantly suppressed by SCH23390 (Fig. 5b). SCH23390 maintained NaPi-2a expression at the apical membrane of PTECs at 10 min (Fig. 5c). Ccr was not different among the five groups (Supplementary Fig. S6b online).

### The decline in serum phosphate levels between 5 and 10 min in the IVC group depends on hepatic phosphate uptake

Phosphate administered through the inferior vena cava circulates and reaches the liver. Since there was no difference between the IVC and PV groups in ΔFEPi between 0–10 min, urinary phosphate excretion during the 10-min period, and renal NaPi-2a expression at 10 min (Fig. 4), we examined the kinetics of phosphate administered through the inferior vena cava. The urinary phosphate excretion in the first 10 min was only approximately 8% of the injected phosphate (Fig. 4b). Therefore, increased phosphaturia could not fully explain how serum phosphate decreased in the IVC group during 5–10 min (P = 0.0188 by the two-tailed Student’s *t-*test) (Fig. 2b). We evaluated the uptake of ^32^P in various organs in the IVC-^32^P and PV-^32^P groups at 10 min, in addition to the evaluation at 5 min in Fig. 3b (Supplementary Fig. S7 online). The ^32^P levels of the liver of the IVC-^32^P group at 10 min were significantly higher than those at 5 min. Moreover, the difference in ^32^P levels in the liver between the IVC-^32^P and PV-^32^P groups at 5 min disappeared at 10 min. The other organs showed no change or decline in the ^32^P levels from 5 to 10 min.

### Phosphorylation of endothelial nitric oxide synthase (eNOS) and levels of phosphate incorporation to CPPs were suppressed in the PV group

**Figure 6:**
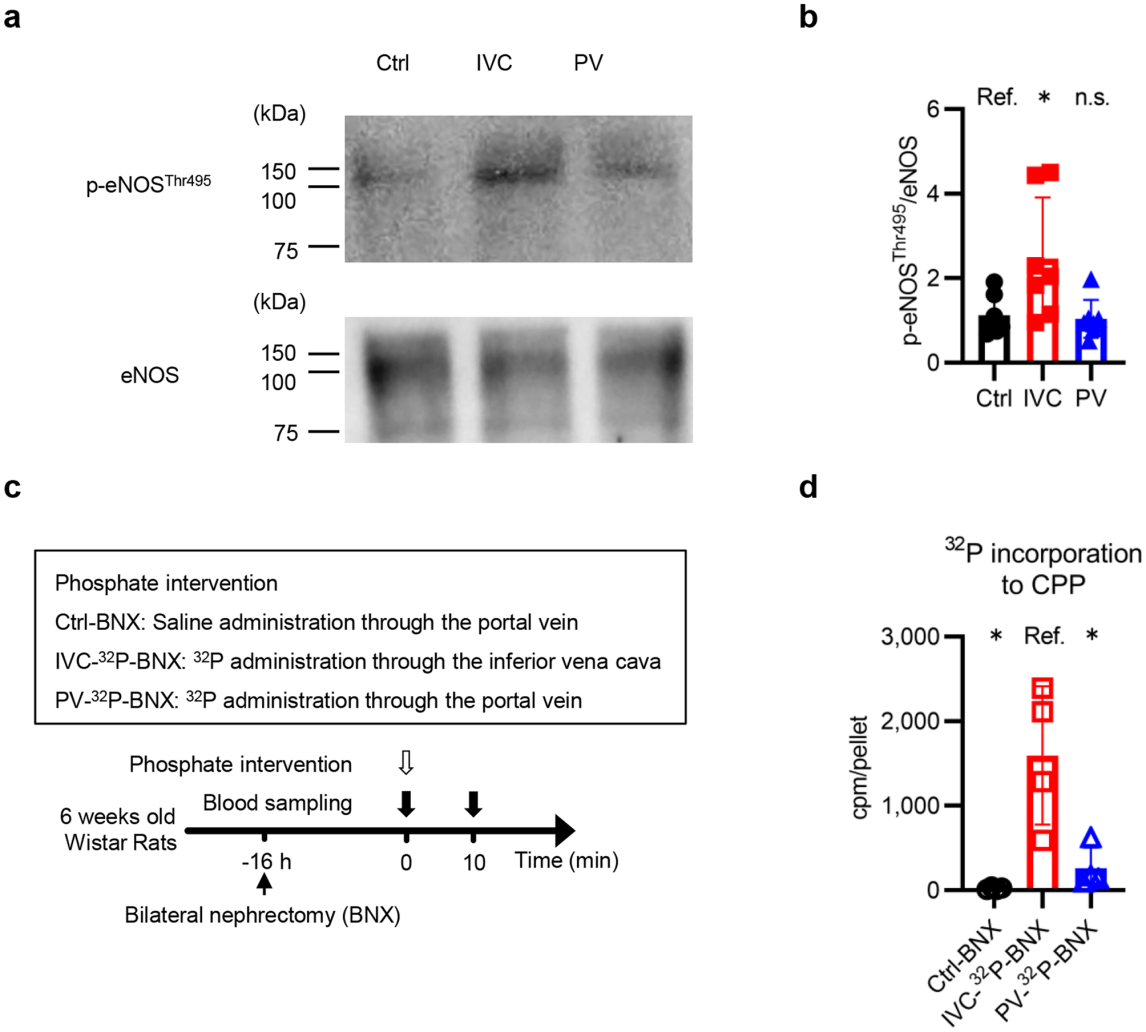
Phosphorylation of endothelial nitric oxide synthase (eNOS) and levels of phosphate incorporation to calciprotein particles (CPPs) were suppressed in the PV group. (a) Representative immunoblots of phosphorylated eNOS (Thr495) and total eNOS in the aorta. Aortic tissues were collected at 20 min in the rats shown in Fig. 2a. The original blots are presented in Supplementary Fig. S11. (**b**) Quantifying phosphorylated eNOS (Thr495) (n = 7 rats per group). (**c**) Experimental design of (**d**). Levels of phosphate incorporation to CPPs were analyzed. BNX rats were randomly divided into three groups, Ctrl-BNX, IVC-^32^P-BNX, and PV-^32^P-BNX. Sixteen hours after the BNX operation, rats in the Ctrl-BNX group were similarly administered normal saline to the rats in Ctrl group. Rats in the IVC-^32^P-BNX and PV-^32^P-BNX groups were administered a radiolabeled phosphate solution similar to those in IVC-^32^P and PV-^32^P groups. At 10 min, blood samples were collected from the inferior vena cava. After the separation of serum (1200 × g for 15 min), levels of ^32^P in the pellet fraction (16,000 × g for 2 h) obtained from the serum were measured (n = 4 rats per group). All results are presented as the means ± SDs, n.s.: not significant, Ref: reference. *P < 0.05 based on Dunnett’s test in (**b**) and (**d**).

We examined the effects of the temporarily elevated serum phosphate concentrations in the IVC group by assessing eNOS phosphorylation (Thr495), which is associated with endothelial dysfunction^20^. Western blot analyses of the aortic tissues collected at 20 min showed that phosphorylation of eNOS increased in the IVC group but not in the PV group (Fig. 6a,b). We also examined levels of phosphate incorporation into CPPs. BNX rats were randomly divided into Ctrl-BNX, IVC-^32^P-BNX, and PV-^32^P-BNX groups, and then similarly received phosphate interventions to the Ctrl, IVC-^32^P, and PV-^32^P groups (Fig. 6c,d). The levels of ^32^P in the CPPs at 10 min from the IVC-^32^P-BNX group were significantly higher than those from the PV-^32^P-BNX group.

### The renal and hepatic nerves play critical roles in the elevation of FEPi between 0–10 min and urinary phosphate excretion during the 10-min period

**Figure 7:**
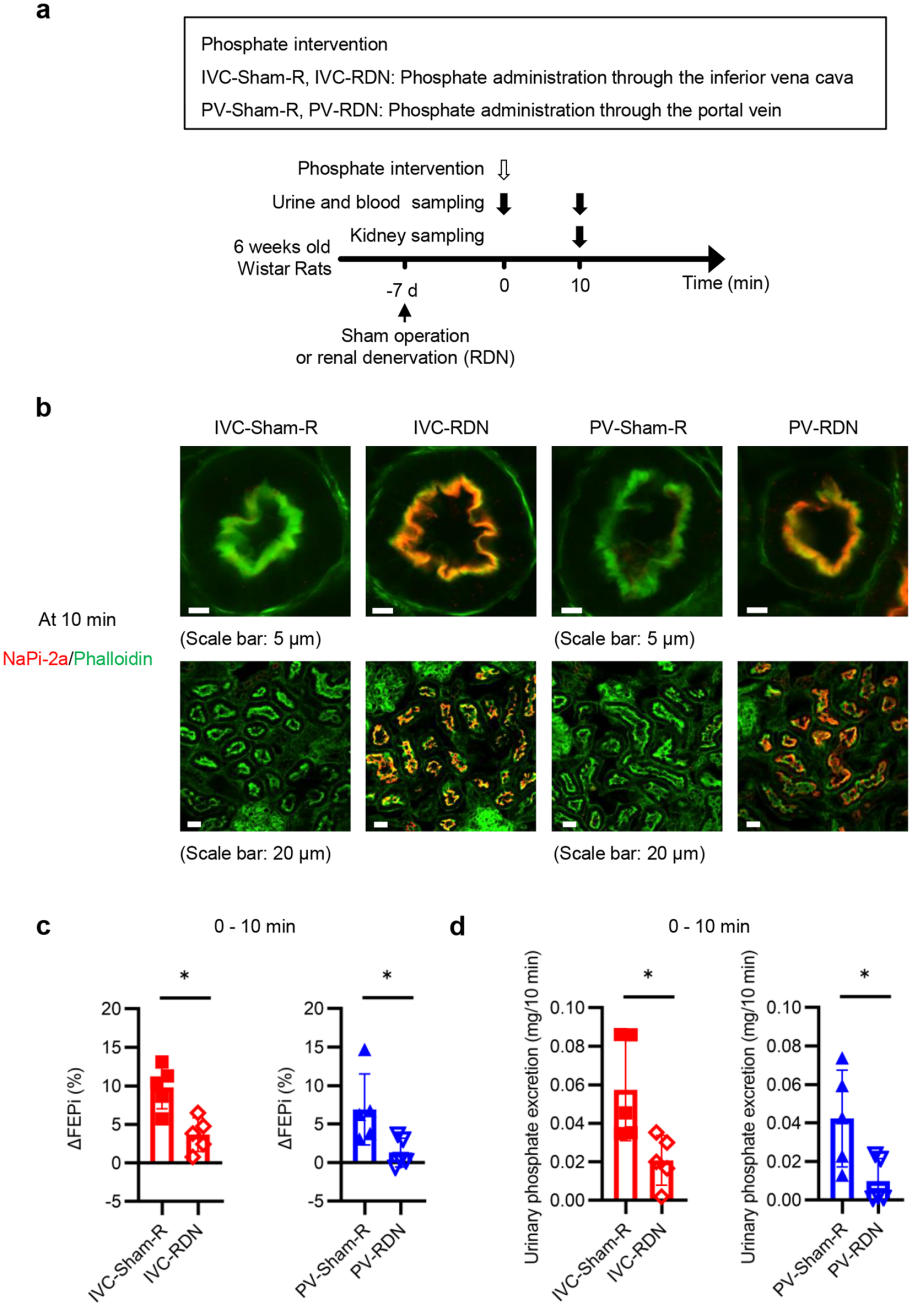
The renal sympathetic nerves played critical roles in the elevation of FEPi between 0 and 10 min and urinary phosphate excretion during the 10-min period. (**a**) Experimental design of (**b**) to (**d**). Sham-operated rats and renal-denervated (RDN) rats were randomly divided into four groups, IVC-Sham-R, PV-Sham-R, IVC-RDN, and PV-RDN. Seven days after the operation, rats in the IVC-sham-R and IVC-RDN groups and in the PV-Sham-R and PV-RDN groups received phosphate interventions similar to those in the IVC and PV groups, respectively. (**b**) Representative immunofluorescence images of renal PTECs stained with phalloidin (green) and NaPi-2a (red) at 10 min. The upper and the lower images in each group are the immunofluorescence images with higher and lower magnification, respectively (scale bars: 5 µm or 20 µm). (c) ΔFEPi between 0 and 10 min (n = 5 rats per group). (**d**) Urinary phosphate excretion during the 10-min period (n = 5 rats per group). All results are presented as the means ± SDs, n.s.: not significant. *P < 0.05 based on the two-tailed Student’s *t-*test in (**c**) and (**d**).

**Figure 8:**
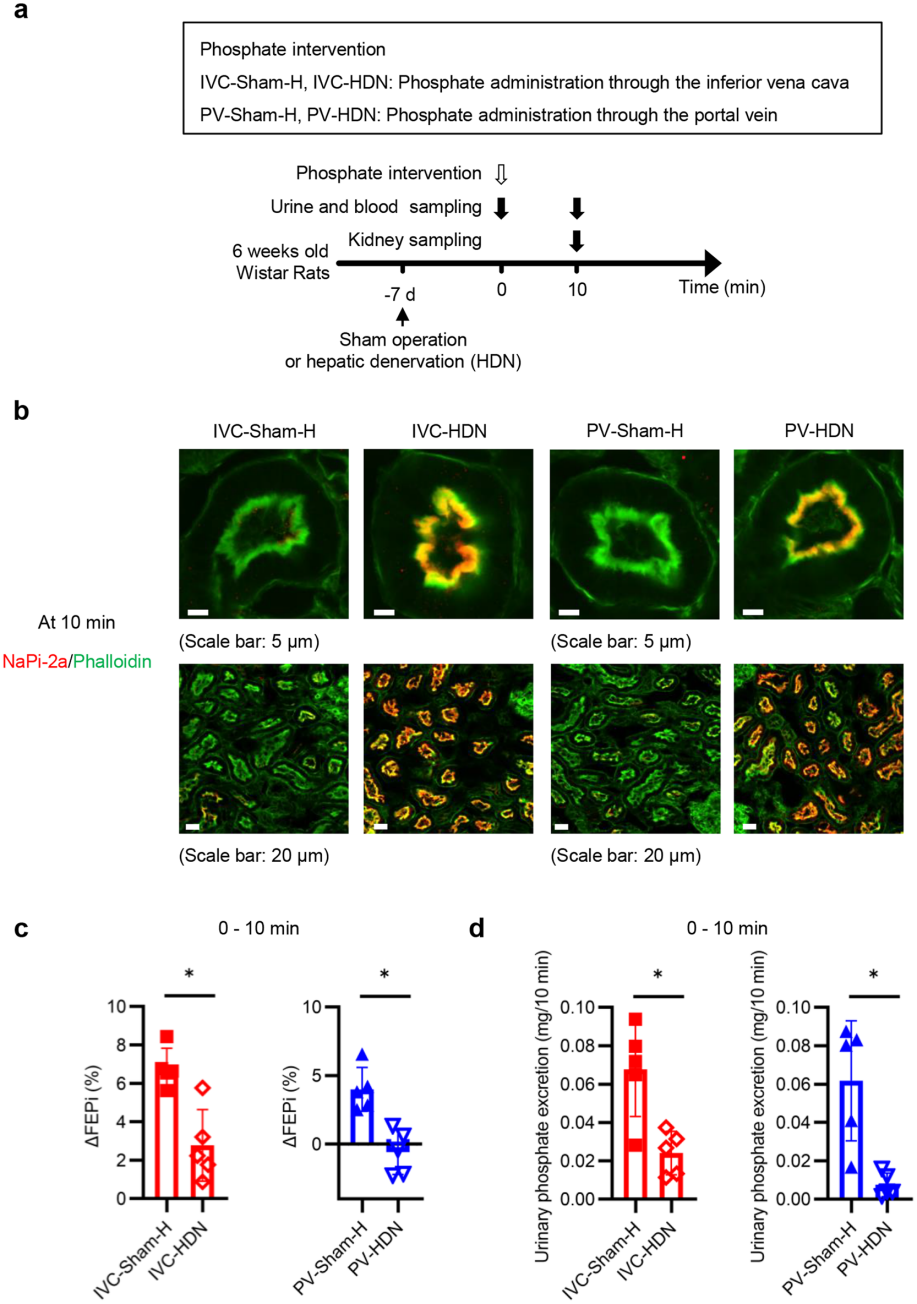
The hepatic nerves played critical roles in the elevation of FEPi between 0 and 10 min and urinary phosphate excretion during the 10-min period. (**a**) Experimental design for (**b**) to (**d**). Sham-operated rats and hepatic-denervated (HDN) rats were randomly divided into four groups, IVC-Sham-H, PV-Sham-H, IVC-HDN, and PV-HDN. Seven days after the operation, rats in the IVC-Sham-H and IVC-HDN groups, and rats in the PV-Sham-H and PV-HDN groups received phosphate interventions similar to those in the IVC and PV groups, respectively. (**b**) Representative immunofluorescence images of renal PTECs stained with phalloidin (green) and NaPi-2a (red) at 10 min. The upper and the lower images in each group are the immunofluorescence images with higher and lower magnification, respectively (scale bars: 5 µm or 20 µm). (**c**) ΔFEPi between 0 and 10 min (n = 5 rats per group). (**d**) Urinary phosphate excretion during the 10-min period (n = 5 rats per group). All results are presented as the means ± SDs, n.s.: not significant. *P < 0.05 based on the two-tailed Student’s *t-*test in (**c**) and (**d**).

Because of the importance of D1-like receptor signaling for the increased phosphaturia during the 10-min period, we investigated renal dopamine decarboxylase (DDC), the rate-limiting enzyme necessary to synthesize dopamine. There was no difference in renal DDC levels in the renal cortex among the Ctrl, IVC, and PV groups (Supplementary Fig. S8 online). Next, we investigated the roles of the nervous system because dopamine is a neurotransmitter. To clarify the role of renal sympathetic nerves, sham-operated and RDN rats were randomly divided into four groups as shown in Fig. 7a. Suppressed norepinephrine levels in the kidney confirmed successful RDN (Supplementary Fig. S9a online). The RDN procedure maintained NaPi-2a expression at the apical membrane of the PTEC (Fig. 7b). FEPi at 10 min was higher than that at 0 min in the sham operated two groups but not in the PV-RDN group (Supplementary Fig. S9b,c online). Although FEPi at 10 min increased in the IVC-RDN group, the magnitude of the increase in FEPi and the urinary phosphate excretion were significantly suppressed by RDN procedure (Fig. 7c,d and Supplementary Fig. S9b). The phosphate intervention following RDN did not affect Ccr (Supplementary Fig. S9d,e online). Because hepatic uptake was the primary determinant of serum phosphate levels in this phase, serum phosphate levels were not affected by RDN (Supplementary Fig. S9f,g online). We also tested the roles of the hepatic nerve using rats with HDN (Fig. 8a). Successful HDN was confirmed in Supplementary Fig. S10a online. HDN showed effects similar to those of RDN (Fig. 8b–d and Supplementary Fig. S10b–g online). These results indicated that the hepatic phosphate uptake activated the hepatic nerve, which was projected to the kidney via the renal sympathetic nerve. This increased urinary phosphate excretion by suppressing NaPi-2a expression levels.

## Discussion

In this study, we investigated the mechanism of acute phosphate regulation by administering a moderate dose of phosphate. Experiments in which phosphate was administered through the portal vein revealed that phosphate could suppress the expression of NaPi-2a and promote phosphaturia independent of changes in serum phosphate, calcium, 1,25VD, plasma i-PTH, and i-FGF23 concentrations. The FEPi increased 10 min after the phosphate administration. However, the amount of urinary phosphate excretion during the period was approximately 8% of the dose, indicating that some organs have taken phosphate before excreted into the urine. Our experiments revealed that hepatic phosphate uptake is critical for maintaining serum phosphate levels. The hepatic uptake increased until 4 h after the duodenal phosphate administration and then turned to decrease. These results indicate that the liver is one of the phosphate reservoirs before cumulative urinary phosphate excretion reaches an amount equivalent to the phosphate load. Interorgan communication between the liver and kidney through the nervous system is pivotal in regulating urinary phosphate excretion.

Thomas et al. demonstrated that intravenous phosphate administration rapidly increases plasma i-PTH concentration and enhances phosphaturia within 10 min. Still, the abundance of NaPi-2a in the brush border membrane (BBM) was unchanged at 40 min in rats^14^. It has been reported that NaPi-2a removal from the BBM is detectable within 5 min after PTH injection in rats^21^. In the current study, we found that the apical expression of NaPi-2a was reduced within 10 min. NaPi-2a under the experimental conditions previously performed by Thomas et al. was refractory to PTH-stimuli^14^. In contrast to their report, we performed our experiments using rats with ad libitum access to a regular diet without fasting. The difference in NaPi-2a response may be due to differences in dietary conditions.

The presence of an intestine-derived phosphaturic factor has been postulated by Berndt et al.^13^ Although they concluded that renal nerves were not responsible for the increase in FEPi, they performed their experiments with rats in which only the left kidney was denervated. In contrast, we performed our experiments on bilateral renal denervation rats. The difference in denervation sites may explain why Berndt et al. did not find a renal nerve-mediated mechanism to promote phosphaturia.

The liver plays an essential role in glucose metabolism. In the fed state, hepatocytes uptake glucose via glucose transporter type 2 and synthesize glycogen and fatty acids^22,23^. Synthesized glycogen is stored in the hepatocyte, while fatty acids are packed into very low-density lipoprotein and delivered to adipose tissues for storage^22,23^. The relationship between the liver and bone in phosphate metabolism may be similar to the relationship between the liver and adipose tissues in glucose metabolism in that the liver is responsible for the temporal storage of phosphate and the bone for long-term storage. However, further research is needed to establish such a concept firmly. Polyphosphate may be a candidate storage form of phosphate, but it is currently unknown whether polyphosphate acts as a storage form of phosphate, like glycogen for glucose, in the liver^24^.

Several points should be acknowledged as limitations. First, transporters responsible for the phosphate uptake by the hepatocytes remain unknown. It also remains obscure how the phosphate taken up by hepatocytes stimulates the nerves. Second, only short-term surrogate markers (i.e., ^32^P uptake into CPPs and phosphorylation of eNOS) were used to assess the significance of phosphate uptake by the liver. The long-term importance of phosphate uptake by the liver remains unclear. Third, we have only shown changes in NaPi2a and have not shown changes in other phosphate transporters (e.g. NaPi2c and sodium-dependent phosphate transporter (Pit)). These points should be addressed in future studies.

In conclusion, our study demonstrates previously unknown mechanisms for maintaining serum phosphate concentration and urinary phosphate excretion. The liver acts as a phosphate reservoir, and the nerve-mediated hepato-renal axis plays an essential role in phosphate homeostasis.

## Methods

### Animal experiments

Six-week-old male Wistar rats were purchased from Japan SLC (Hamamatsu, Japan). For all experiments, rats aged 6–8 weeks were used. All the rats had ad libitum access to water and a regular chow diet containing 1.07% calcium and 0.83% phosphorus (MF diet, Oriental Yeast, Tokyo, Japan). All animal experiments were done in the morning without fasting. When performing invasive procedures, the rats were anesthetized with an intraperitoneal injection of medetomidine, midazolam, and butorphanol and were placed on a heated table to maintain body temperature between 36 and 38 °C. The Animal Committee of Osaka University approved all experiments (approval number: 01-046-005), and the study was performed under standard guidelines regarding the use of animals in scientific experiments and was reported in accordance with ARRIVE guidelines.

### Administration of radiolabeled sodium phosphate through the duodenum

For Duo group rats, a 24-gauge catheter (SR-FS2419, TERUMO, Tokyo, Japan) was inserted into the duodenum 1 cm anorectally from the gastric pylorus under open surgery. A Na_2_HPO_4_/NaH_2_PO_4_ solution (phosphate 80 mM, sodium 154 mM, pH 7.4, 1.0 ml/animal) containing trace amounts of radiolabeled phosphate (^32^P) (1.0 × 10^–5^ µmol/ml, 3700 Bq/ml, Perkin Elmer, MA, USA) was administered through the catheter. To prevent dehydration, the surgical wound was covered with gauze soaked in saline solution warmed to 36–38 °C. Blood and tissue samples were solubilized and color quenched by incubating them in a solution containing 1.0 ml of 60% perchloric acid (Nacalai Tesque, Kyoto, Japan) and 0.4 ml of 10% hydrogen peroxide (Wako) at 60 °C for 3 h. The levels of ^32^P were measured using Cerenkov counting (AccuFLEX LSC 7400, Hitachi Health care Manufacturing, Chiba, Japan).

### Sodium phosphate administration through the portal vein or inferior vena cava

Effects of phosphate administration through the portal vein or inferior vena cava (referred to as phosphate intervention) were evaluated. To access blood vessels for the phosphate injections, a catheter (SR-FS2419) was inserted into the inferior vena cava or portal vein. To avoid dehydration, normal saline (1.0 ml/animal) was injected before the phosphate interventions. The surgical wound was covered similarly to the Duo group. If group names do not contain ^32^P, a Na_2_HPO_4_/NaH_2_PO_4_ solution (phosphate 20 mM, sodium 154 mM, pH 7.4, 1.0 ml/animal) was administered through the inferior vena cava and portal vein in the groups whose group name contains IVC and groups whose group name contains PV, except for the PV-100 and PV-500 groups, respectively. Normal saline (1.0 m/animal) was administered through the portal vein in the groups whose group name contains Ctrl. A Na_2_HPO_4_/NaH_2_PO_4_ solution containing ^32^P (phosphate 20 mM, ^32^P 3700 Bq/ml, sodium 154 mM, pH 7.4, 1.0 ml/animal) was administered in the groups whose group name contains IVC-^32^P and the groups whose group name contains PV-^32^P similarly as in the IVC and PV groups, respectively. Blood samples were collected from the inferior vena cava.

### Blood and urine collection procedures

For the Duo group, catheters (SR-FS2419) were inserted into the inferior vena cava and portal vein to collect blood samples. For the other groups, i.e., those receiving phosphate interventions through blood vessels, a 24-gauge catheter was inserted into the inferior vena cava to collect blood samples. At the indicated time points, 0.1 ml of blood was discarded, followed by 0.1 ml of blood collection. After each blood collection procedure, 0.2 ml of saline was administered through the catheters. Blood samples were centrifuged in heparin tubes for 15 min at 1200 × g to obtain plasma samples. To obtain serum samples, blood samples were allowed to clot at room temperature and then centrifuged for 15 min at 1200 × g. Urine samples were collected by puncturing the bladder, followed by centrifugation for 15 min at 1200 × g. Plasma, serum, and urine samples were stored at − 80 °C until analysis. FEPi was calculated using the formula: (urine phosphate (mg/dl) × serum creatinine (mg/dl)) × 100/(serum phosphate (mg/dl) × urine creatinine (mg/dl)). Ccr was calculated using the formula: (urine creatinine (md/dl) × urine volume (ml/min)) / serum creatinine (mg/dl).

### Pretreatments

Rats with various pretreatments received the phosphate interventions. Groups were named according to the combination of pretreatment and phosphate intervention types. The details of each group's intervention are summarized in Supplementary Table S1 online.

A bilateral nephrectomy (BNX) was performed by removing both kidneys after ligating the renal artery, renal vein, and urinary tract. Partial hepatectomy (PH) was achieved by surgically removing the median and left lateral lobes. A Kupffer cell depletion model or sinusoidal endothelial cell dysfunction model was prepared by intraperitoneally injecting gadolinium chloride (GdCl_3_) (40 mg/kg, Sigma‒Aldrich) or monocrotaline (MCT) (200 mg/kg, Sigma‒Aldrich) dissolved in 1.0 ml of normal saline, respectively. To inhibit D1-like dopamine receptor, SCH23390 dissolved in 1.0 ml of normal saline was intraperitoneally injected (500 µg/kg BW, Sigma‒Aldrich). Vehicle-pretreated rats received intraperitoneal injections of normal saline (1.0 ml/animal). Bilateral renal denervation (RDN) was performed by surgical denervation with a topical application of 10% phenol dissolved in ethanol. Hepatic nerves were ablated (HDN rats) by applying a 10% phenol solution to the hepatic artery, portal vein, and common bile duct using a cotton swab.

### Biochemical measurement

Levels of phosphate, calcium, and creatinine were measured using each assay kit (Wako, Osaka, Japan). Plasma i-PTH and i-FGF23 concentrations were measured using Rat BioActive Intact PTH ELISA (Immutopics, CA, USA) and FGF23 ELISA kits (Kainos, Tokyo, Japan), respectively. Serum samples were sent to SRL (Tokyo, Japan) to measure serum 1,25VD levels.

### Antibodies

Antibodies against specific molecules were obtained as follows: CD68 (catalog: ab25212, Abcam Cambridge, UK), hepatic sinusoid endothelial cells (SE-1) (catalog: NB110-68095, Novusbio, Colorado, USA), Alexa Fluor 555 donkey anti-rabbit IgG antibody (catalog: A-31572, Invitrogen), endothelial nitric oxide synthase (eNOS) (catalog: #9572, Cell Signaling, Danvers, USA), phospho-eNOS Thr495 (catalog: #9574, Cell Signaling), and horseradish peroxidase (HRP)-conjugated 2^nd^ Ab for WB (catalog: P0448, DAKO, Santa Clara, USA).

A polyclonal antibody against sodium-phosphate cotransporter 2a (NaPi-2a) was raised in rabbits by immunization with rat NaPi-2a peptide (MMSYSERLGGPAVSP)^25^.

### Histology and immunostaining

Tissues were fixed with 4.0% paraformaldehyde in phosphate-buffered saline (PBS) (pH 7.4) for 6 h, cryoprotected with 30% sucrose in PBS for two days, and then embedded in optimal cutting temperature compound (Sakura, Tokyo, Japan). Freshly prepared 0.1% sodium borohydride (catalog: 452882-5G, Sigma‒Aldrich, MO, USA) in Tris-buffered saline (TBS) was applied for 30 min to reduce background autofluorescence as needed. Tissue sections (10 µm) were incubated with a blocking solution (1.5% horse serum (Vector laboratories, California, USA) in PBS or 3% bovine serum (Sigma‒Aldrich, MO, USA) in PBS) followed by primary antibody reactions overnight at 4 °C. The sections were washed three times in PBS and then incubated with Alexa Fluor 555-conjugated secondary antibodies at room temperature for 1 h. FITC-phalloidin (Invitrogen, 0.066 µM) was added to the secondary antibody solution where indicated. Again, the sections were washed three times in PBS and mounted with VectaShield (Vector Laboratories, California, USA). The dilution ratios of the primary and secondary antibodies were as follows: CD68 1:150, SE-1 1:150, NaPi-2a 1:200, and Alexa Fluor 555 1:200. Immunofluorescence images were obtained using confocal microscopy (FV-1000D or SpinSR10, Olympus, Tokyo, Japan).

### Western blot analyses

Proteins were extracted from the aorta using the RIPA Lysis Buffer system (Santa Cruz, Texas, USA). Extracted protein samples were diluted in 2 × electrophoresis sample buffer (Santa Cruz) containing dithiothreitol. Each sample was subjected to sodium dodecyl sulfate–polyacrylamide gel electrophoresis (SDS‒PAGE) using a 10% acrylamide gel and electroblotted onto a polyvinylidene difluoride (PVDF) membrane (Amersham, Germany)^26^. The membrane was cut prior to hybridization to reduce the amount of antibodies used. Then, the membrane was incubated with a blocking solution (5% bovine serum albumin (BSA) in TBS containing 0.1% Tween 20 (TBS-T)) followed by anti-eNOS or anti-phospho-eNOS Thr495 antibody reactions overnight at 4 °C. The membranes were washed three times in TBS-T and then incubated with secondary antibodies at room temperature for 1 h. Again, the membranes were washed three times in TBS-T. Signals were detected by enhanced chemiluminescent reagents (Bio-Rad Laboratories). Images were obtained by using ChemiDoc Touch (Bio-Rad Laboratory). The dilution ratios of antibodies were as follows: eNOS 1:1000, phospho-eNOS Thr495, 1:1000, and HRP secondary antibody 1:3000. Full immunoblot images are shown in Supplementary Figs. S11–S12 online.

### Analysis of calciprotein particles (CPPs)

To measure ^32^P incorporation levels to the CPPs in the BNX rats, serum was processed as described previously^27^. Fifty-microliter aliquots of serum in 1.5-ml tubes were centrifuged at room temperature at 16,000 × g for 2 h. After discarding the supernatant, each tube was rinsed with 100 µl of 150 mM NaCl solution and centrifuged for 5 min at 16,000 g. The resultant pellets were solubilized, and the color was quenched in the same way as above. The levels of ^32^P in the resultant solutions were measured using Cerenkov counting (AccuFLEX LSC 740).

### Statistical analysis

A *t*-test evaluated significant differences between two groups. Multiple-group comparisons were evaluated using Dunnett’s test. Repeated measures of ANOVA were applied where indicated. Statistical analyses were performed using GraphPad Prism 8.0 software (GraphPad Software, San Diego, CA, USA) or JMP 16.1 software (SAS Institute, Cary, NC, USA). A *P* value less than 0.05 was considered statistically significant. Bonferroni correction was applied for multiple comparisons where indicated.

## Supplementary Information

Supplementary Information.

## Data Availability

This study did not generate large biological datasets that require a deposit in public repositories. The datasets used and/or analyzed during the current study available from the corresponding author on reasonable request.
