# Supplementary material for: Hepatic phosphate uptake and subsequent nerve-mediated phosphaturia are crucial for phosphate homeostasis following portal vein passage of phosphate in rats
Source: Sci Rep. 2023 Apr 8;13:5794. doi: 10.1038/s41598-023-32856-2 (PMC10082792; doi:10.1038/s41598-023-32856-2)
Supplement: Supplementary file 1 — Supplementary Information. [file 41598_2023_32856_MOESM1_ESM.pdf]

Hepatic phosphate uptake and subsequent nerve-mediated phosphaturia are crucial for phosphate homeostasis following portal vein passage of phosphate in rats

Seiichi Yasuda M.D., Kazunori Inoue M.D., Ph.D., Isao Matsui M.D., Ph.D., Ayumi Matsumoto M.D., Ph.D., Yusuke Katsuma M.D., Hiroki Okushima M.D., Atsuhiro Imai M.D., Yusuke Sakaguchi M.D., Ph.D., Jun-ya Kaimori M.D., Ph.D., Ryohei Yamamoto M.D., Ph.D., Masayuki Mizui M.D., Ph.D., and Yoshitaka Isaka M.D., Ph.D.

Supplementary Methods

Pretreatments

Thyroparathyroidectomy (TPTX) was performed by surgically removing the thyroid and parathyroid glands en bloc. Thyroid hormone was supplemented by intraperitoneal injection of L-thyroxine (4 µg/kg body weight, catalog: T2376, Sigma–Aldrich, MO, USA) just after TPTX. A sham operation was performed similarly without surgically removing the thyroid and parathyroid glands. Sham-operated rats served as a control in determining whether TPTX was successful. TPTX rats were randomly divided into three groups, Ctrl-TPTX, IVC-TPTX, and PV-TPTX. Twenty-

four hours after the operation, rats in the Ctrl-TPTX, IVC-TPTX, and PV-TPTX groups similarly received phosphate interventions to the rats in the Ctrl, IVC, and PV groups, respectively.

Quantitative reverse transcription-PCR

RNA extracted using TRIzol (#15596026, Invitrogen, Carlsbad, CA, USA) was converted to single-strand DNA with random primers (#48190011, Invitrogen) and SuperScript II (#18064022 Invitrogen). Each cDNA was mixed with forward and reverse primers and power SYBR green PCR master mix (#4367659, Applied Biosystems, CA, USA) and then analyzed with ABI PRISM 7900HT (Applied Biosystems, MA, USA). The primer sequences were as follows:

Cd68 forward 5'- TCCAGCAATTCACCTGGACC -3',

Cd68 reverse 5'- AAGAGAAGCATGGCCCGAAG -3',

Adgre1 forward 5'- CCACAACACCTACCTGCACC -3',

Adgre1 reverse 5'- GATAGCGCAAGCTGTCTGGTT -3',

Nampt forward 5'- TCTGGCCCGAGATGAATG -3',

Nampt reverse 5'- GGGTGGGTATTGTTTATAGTGAGTAAC -3',

Gapdh forward 5'- GGATGCAGGGATGATGTT -3',

and *Gapdh* reverse 5'- TGCACCACCAACTGCTTA -3'.

Primer sequences were designed using Primer-BLAST (https://www.ncbi.nlm.nih.gov/tools/primer-blast/).

Nicotinamide adenine dinucleotide (NAD) analyses

Rat kidney samples from the Ctrl, IVC, and PV groups were collected at 10 min. For the measurement of NAD in the kidneys, the NAD/NADH Quantification Kit was used according to the manufacturer's instructions (#MAK037, Sigma–Aldrich).

Dopa decarboxylase (DDC) analyses

Rat kidney samples from the Ctrl, IVC, and PV groups were collected at 10 min. The DDC level in the renal cortex was measured using the Rat Dopachrome Decarboxylase ELISA Kit according to the manufacturer's instructions (catalog: MBS730226, MyBioSource, British Columbia, Canada).

Norepinephrine analyses

Rat kidney and liver samples were collected at 20 min. Norepinephrine levels in the kidneys and livers were measured using the Noradrenaline research ELISA kit according to the manufacturer's instructions (catalog: BA-E-5200, ImmuSmol Talence, Bordeaux, France).

Supplementary Figure S1.

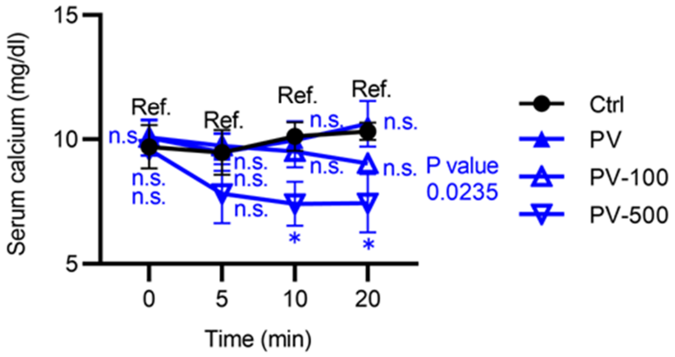

Supplementary Figure S1. Serum calcium levels following different phosphate injection doses into the portal vein.

Normal six-week-old male Wistar rats were randomly divided into four groups, Ctrl, PV, PV-100, and PV-500. The rats in the Crtl (0 μmol phosphate) and PV (20 μmol phosphate) groups received the same phosphate intervention as in Fig. 2a. The rats in the PV-100 and PV-500 groups received 100 μmol and 500 μmol of phosphate at time 0 through the portal vein. Serum samples were collected from the inferior vena cava at the indicated time. Serum calcium levels are shown (n = 5 rats per group). All results are presented as the means ± SDs, n.s.: not significant, Ref.: reference. *$P <$ 0.0166 based on Dunnett's test with Bonferroni correction. P-value = 0.0235 (Ctrl vs PV-100 at 20 min).

Supplementary Figure S2.

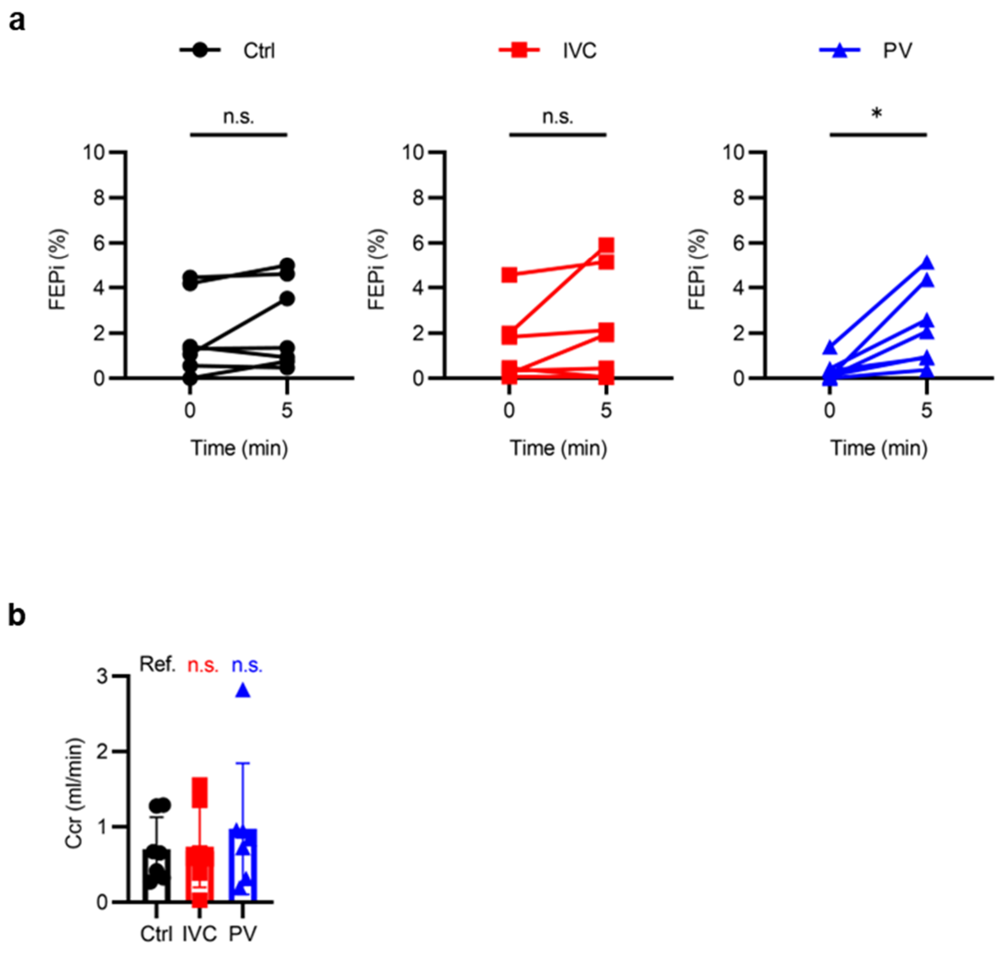

Supplementary Figure S2. FEPi and creatinine clearance (Ccr) of the rats shown in Fig. 2a.

(a) FEPi at 0 and 5 min after the phosphate intervention in each group rat (n = 7 rats per group). The spaghetti plots which connect the repeated measurements of the same rat were shown. (b) Ccr during the first 5 min-period in each group rat (n = 7 rats per group). The result in (b) is presented as the means ± SDs. n.s.: not significant, Ref: reference. *P < 0.05 based on the paired *t*-test in (a) and Dunnett's test in (b).

Supplementary Figure S3.

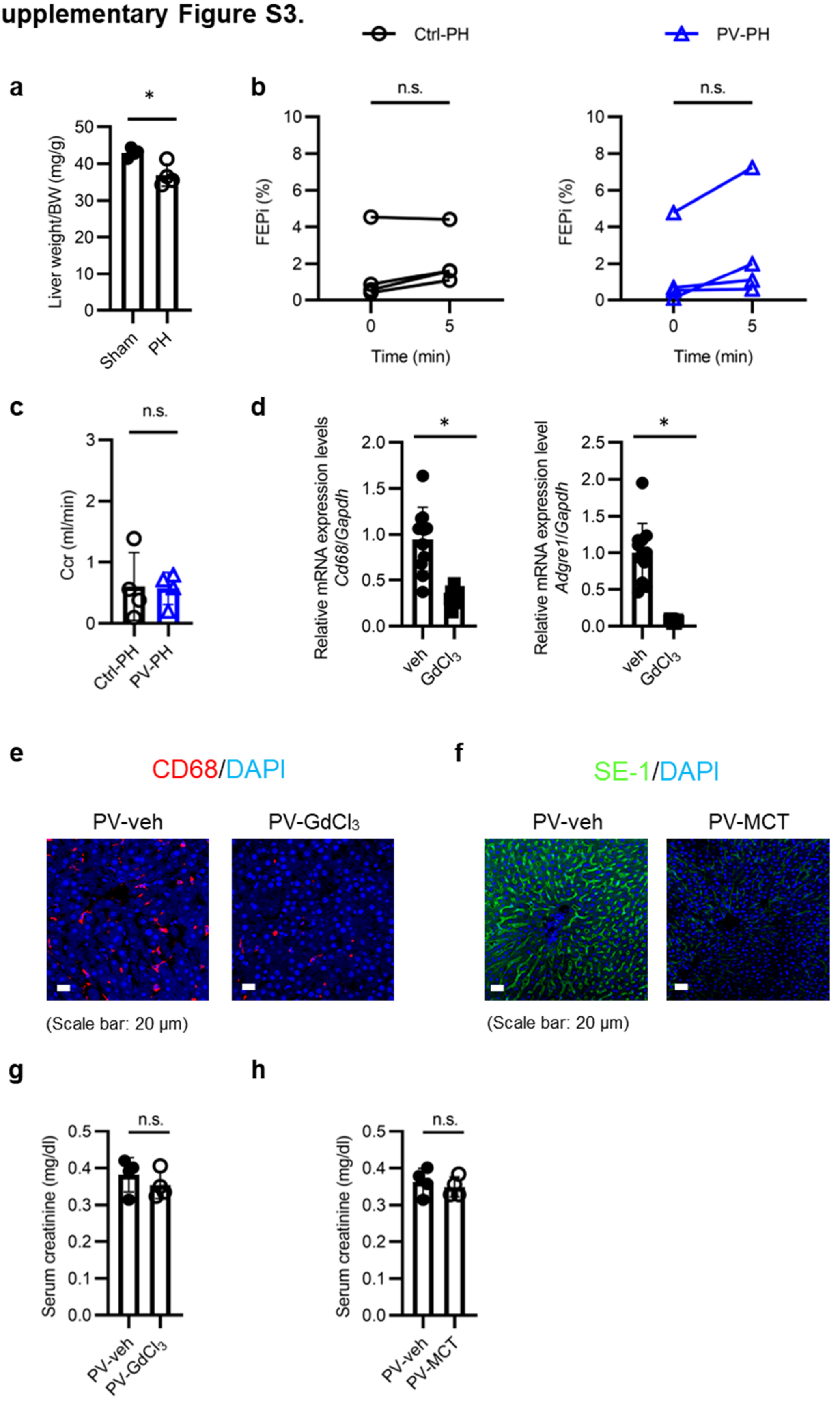

Supplementary Figure S3. Experiments related to partial hepatectomy (PH), Kupffer cells, and sinusoidal endothelial cells

(a) The liver weight/body weight ratios at 70 hours after the sham operation or PH operation in the rats shown in Fig. 3c (n = 4 rats per group). (b) FEPi at 0 and 5 min after the phosphate intervention in the Ctrl-PH and PV-PH groups (n = 4 rats per group). (c) Ccr during the first 5 min-period in each group rat (n = 4 rats per group). (d) Hepatic *Cd68* and *Adgre1* mRNA expression levels in the rats pretreated with vehicle or $GdCl_3$ (n = 12-13 rats per group). Liver samples of the vehicle group were obtained from the PV-veh group shown in Fig. 3f. Liver samples of the $GdCl_3$ group in this figure were a mixture of Ctrl-$GdCl_3$, IVC-$GdCl_3$, and PV-$GdCl_3$ groups shown in Fig. 3f. Samples were collected at 20 min. (e) Representative immunofluorescence images of the liver stained with CD68 (red) in the PV-veh and PV-$GdCl_3$ groups. (f) Representative immunofluorescence images of the liver stained with sinusoidal endothelial cell-1 (SE-1) (green) in the PV-veh and PV-MCT groups. The nuclei were stained with DAPI (blue) (scale bars: 20 µm). Serum creatinine levels of (g) the PV-veh, PV-$GdCl_3$ groups and (h) the PV-veh, PV-MCT groups (n = 4 in each group). The results are presented as the means ± SDs, n.s.: not significant, Ref: reference. *$P < 0.05$ based on the two-tailed Student's *t*-test in (a), (c), (d), (g), and (h), and the paired *t*-test in (b).

Supplementary Figure S4.

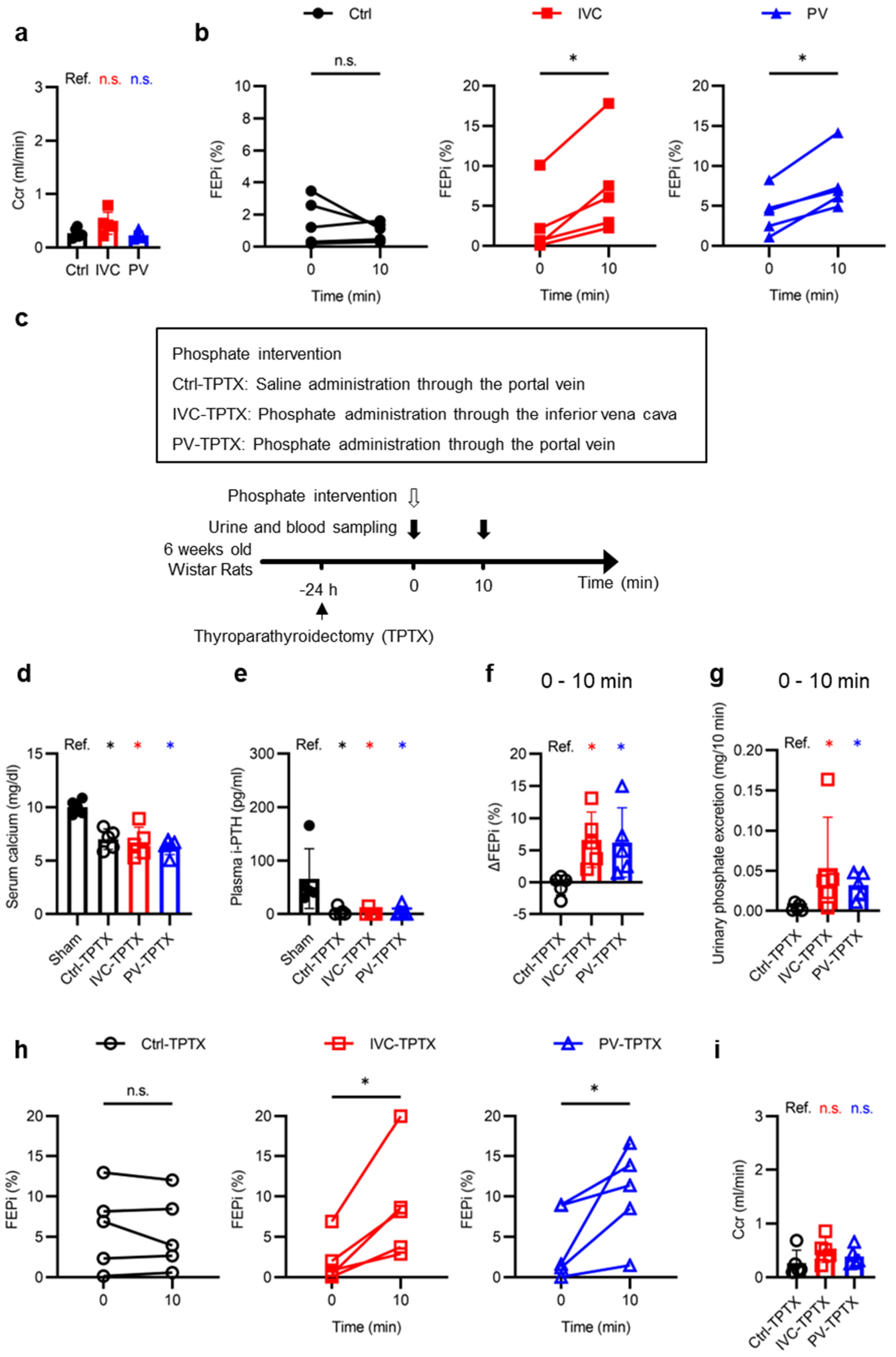

Supplementary Figure S4. Rats in the IVC and PV groups increased FEPi and urinary phosphate excretion within the first 10 minutes in a plasma PTH-independent manner.

(a) Ccr during the first 10 min-period in each group rat shown in Fig. 4a (n = 7 rats per group). (b) FEPi at 0 and 10 min after the phosphate intervention in each group rat shown in Fig. 4a (n = 7 rats per group). (c) Experimental design of (d)-(i). Thyroparathyroidectomized (TPTX)-rats were randomly divided into three groups, Ctrl-TPTX, IVC-TPTX, and PV-TPTX. Twenty-four hours after the operation, rats in the Ctrl-TPTX, IVC-TPTX, and PV-TPTX groups similarly received phosphate interventions to the rats in the Ctrl, IVC, and PV groups, respectively. (d-e) Sham-operated rats served as a control in determining whether TPTX was successful. Levels of (d) serum calcium and (e) plasma i-PTH at 0 min were measured (n = 5 rats per group). (f) ΔFEPi between 0 and 10 min and (g) urinary phosphate excretion during the 10-min period in each group (n = 5 rats per group). (h) FEPi at 0 and 10 min after the phosphate intervention in each group rat with TPTX (n = 5 rats per group). (i) Ccr during the first 10-min period in each group rat with TPTX (n = 5 rats per group). The results are presented as the means ± SDs, n.s.: not significant, Ref.: reference. *$P < 0.05$ based on Dunnett's test in (a), (d) to (g), and (i), and the paired *t*-test in (b) and (h).

Supplementary Figure S5.

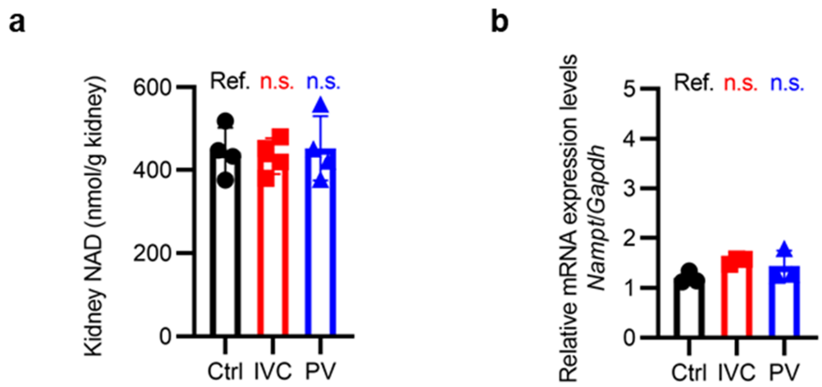

Supplementary Figure S5. Phosphate interventions did not affect renal nicotinamide adenine dinucleotide (NAD) levels.

(a) NAD content of the kidney at 10 min in the treated rats shown in Fig. 4a (n = 4 rats per group). (b) Renal *Nampt* mRNA expression levels at 10 min in the treated rats shown in Fig. 4a (n = 3 rats per group). All results are presented as the means ± SDs, n.s.: not significant, Ref.: reference. **P* < 0.05 based on Dunnett's test.

Supplementary Figure S6.

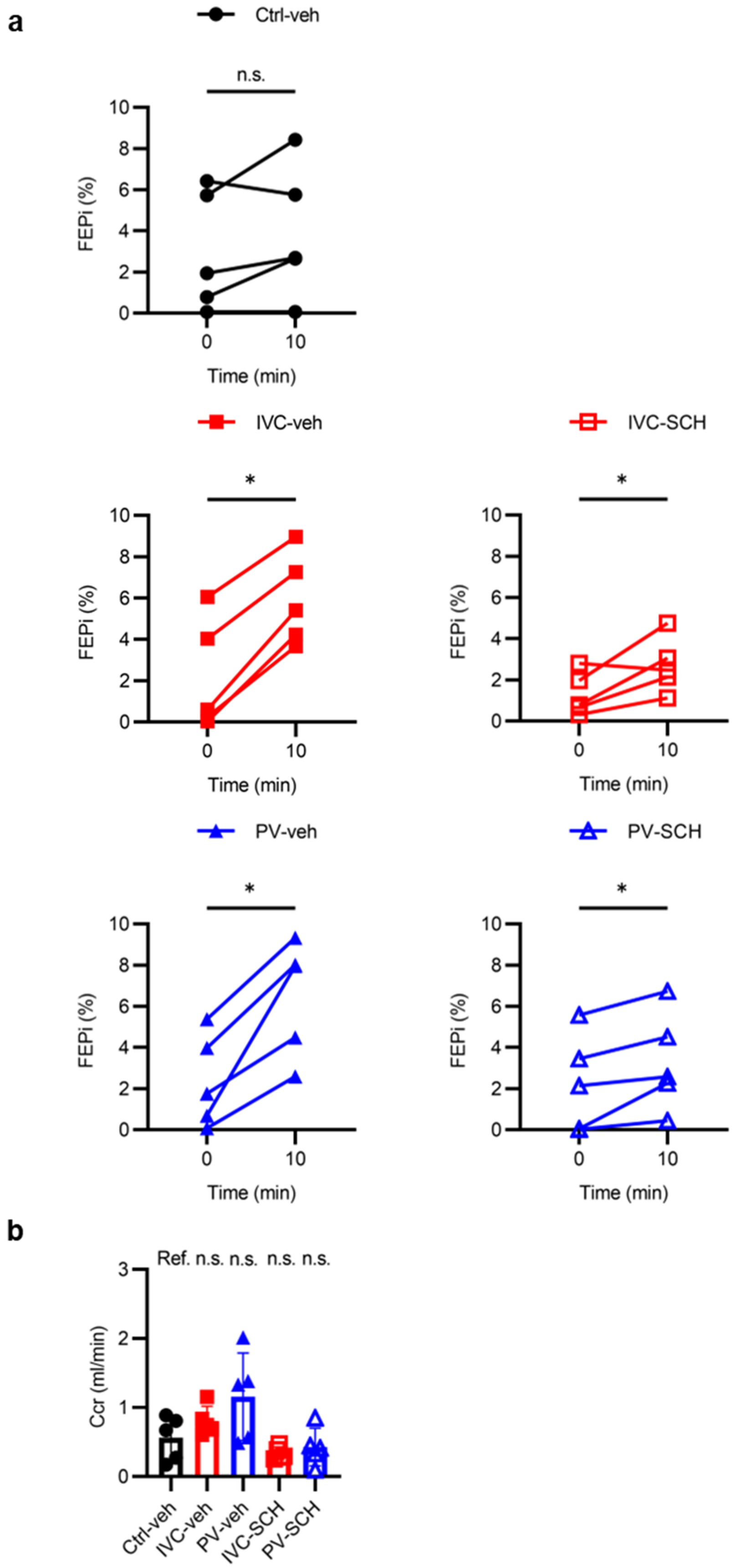

Supplementary Figure S6. FEPi and Ccr levels in the Ctrl-veh, IVC-veh, IVC-SCH, PV-veh, and PV-SCH groups

Parameters of the rats shown in Fig. 5 are summarized. (a) FEPi at 0 and 10 min after the phosphate intervention in each group rat(n = 5 rats per group). (b) Ccr during the first 10-min period in each group rat (n = 5 rats per group). The result in (b) is presented as the means ± SDs. n.s.: not significant, Ref: reference. *$P < 0.05$ based on the paired *t*-test in (a) and Dunnett’s test in (b).

Supplementary Figure S7.

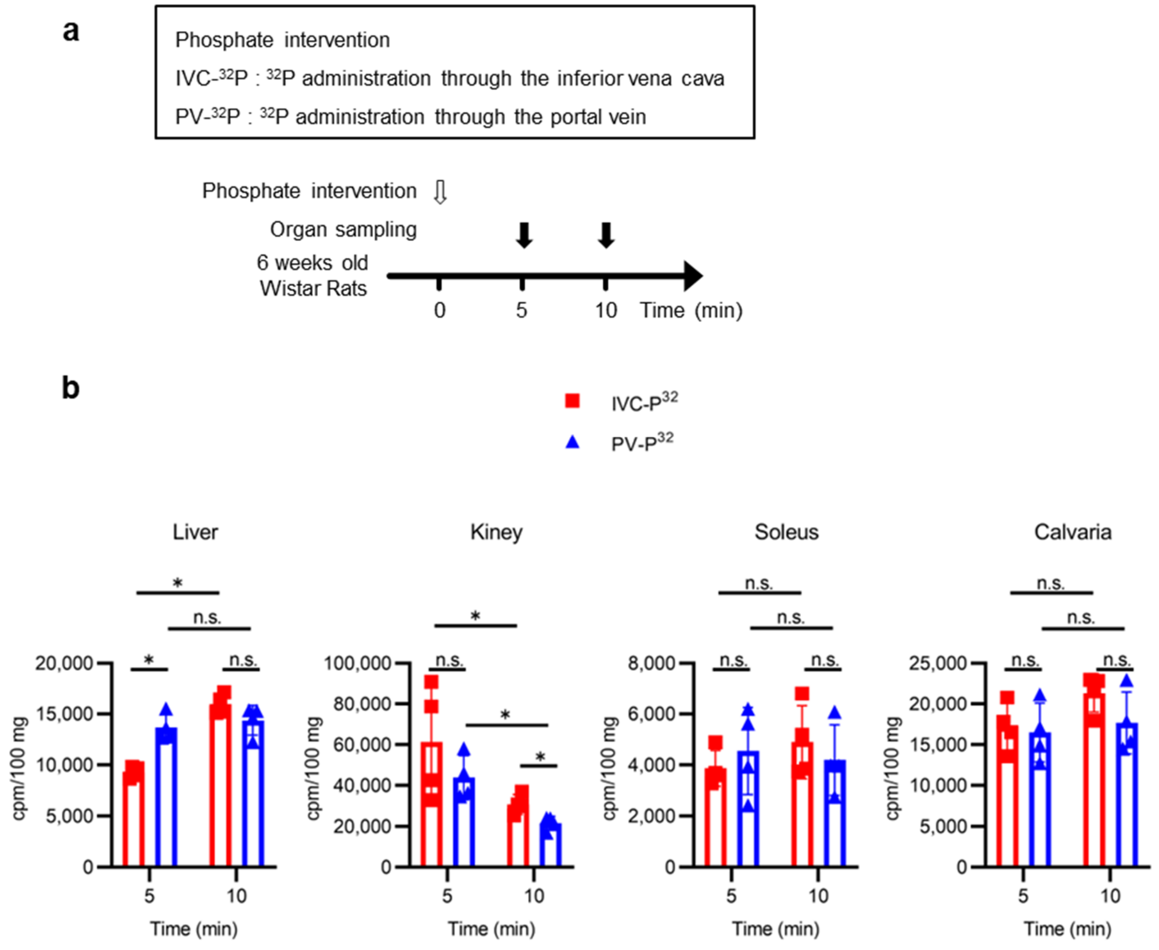

Supplementary Figure S7. Levels of ^{32}P in the liver of the IVC-^{32}P group at 10 min were significantly higher than those at 5 min.

(a) Normal rats were randomly divided into the IVC-^{32}P group and the PV-^{32}P group, as shown in Figure 3a. (b) The levels of ^{32}P in the liver, kidney, soleus, and calvaria at 10 min were analyzed in addition to those at 5 min shown in Figure 3b (n = 4 rats per group). The results are presented as the means ± SDs, n.s.: not significant, Ref.: reference. *P < 0.05 based on the two-tailed Student's *t*-test in (b).

Supplementary Figure S8.

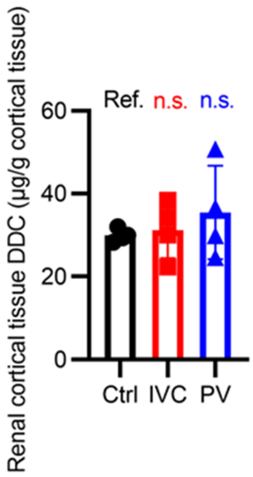

Supplementary Figure S8. Dopa decarboxylase (DDC) levels in the renal cortex did not increase following the phosphate interventions.

DDC levels in the renal cortex at 10 min in the treated rats shown in Fig. 4a (n = 4 rats per group). The results are presented as the means ± SDs and analyzed by Dunnett's test, n.s.: not significant, Ref.: reference.

Supplementary Figure S9.

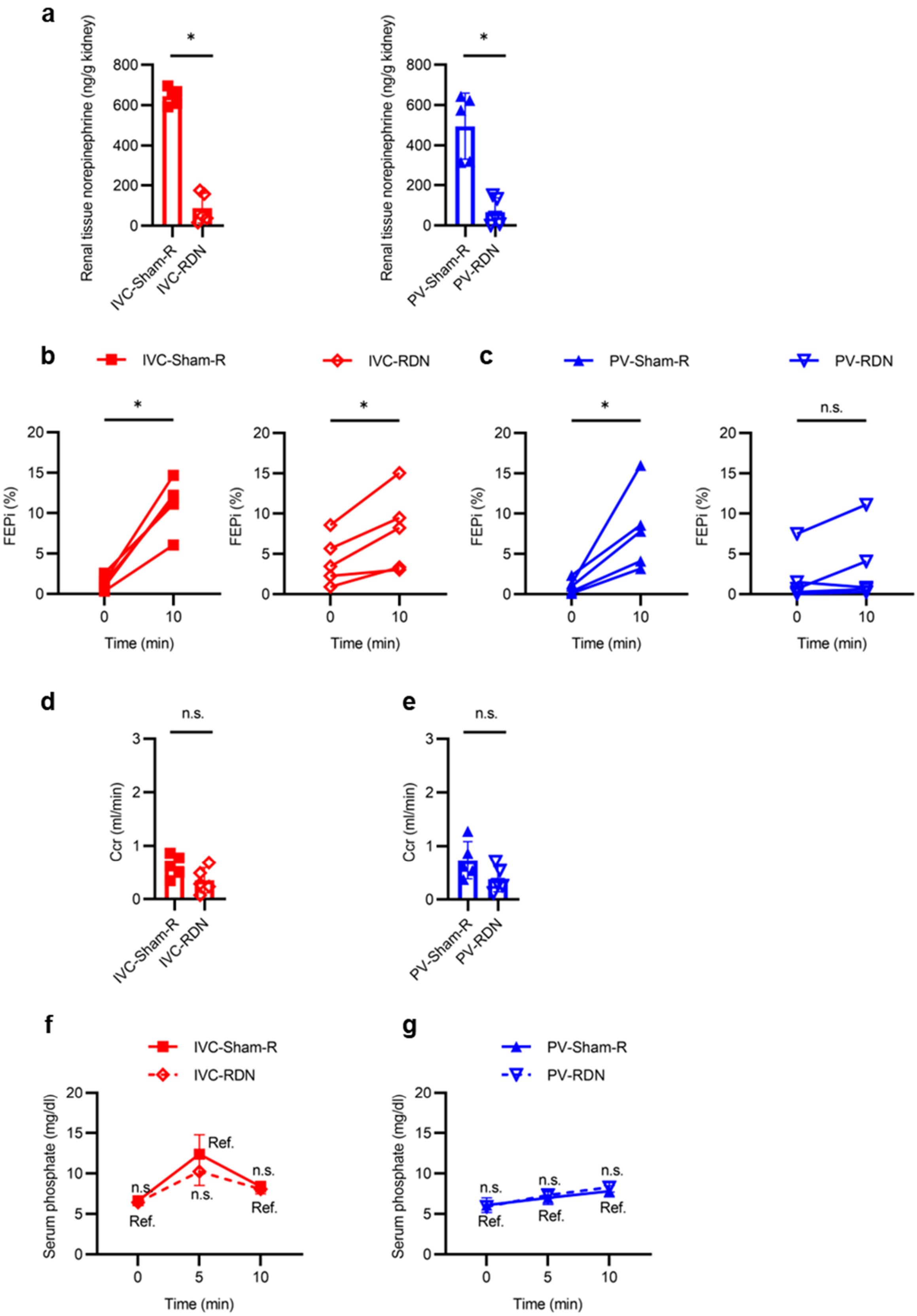

Supplementary Figure S9. Renal nervous system-related experiments.

Parameters of the rats shown in Fig. 7 are summarized. (a) Norepinephrine levels in the rat kidney shown in Fig. 7a (n = 5 rats per group). (b-c) FEPi at 0 and 10 min after the phosphate intervention in each group rat (n = 5 rats per group). (d-e) Ccr during the first 10 min period in each group rat (n = 5 rats per group). (f-g) Serum phosphate concentration (f) in the IVC-Sham-R and IVC-RDN group rats and (g) in the PV-Sham-R and PV-RDN group rats (n = 5 rats per group). The results are presented as the means ± SDs, n.s.: not significant, Ref.: reference. *$P < 0.05$ based on the two-tailed Student's *t*-test in (a), (d), and (e), the paired *t*-test in (b) and (c), and the two-tailed Student's *t*-test with Bonferroni correction in (f) and (g).

Supplementary Figure S10.

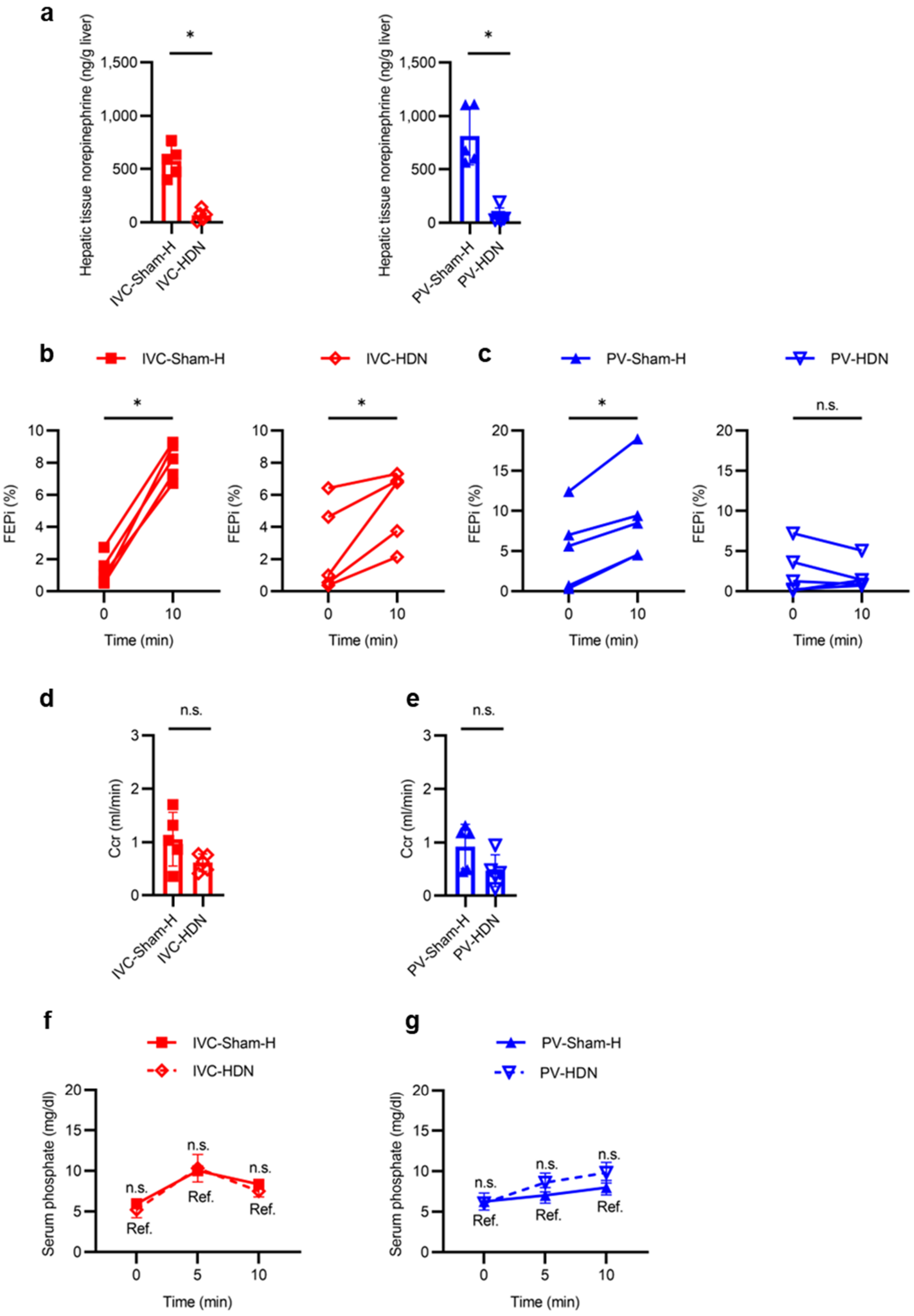

Supplementary Figure S10. Hepatic nervous system-related experiments.

Parameters of the rats shown in Fig. 8 are summarized. (a) Norepinephrine levels in the rat liver shown in Figure 8a (n = 5 rats per group). (b-c) FEPi at 0 and 10 min after the phosphate intervention in each group rat (n = 5 rats per group). (d-e) Ccr during the first 10-min period in each group rat (n = 5 rats per group). (f-g) Serum phosphate concentration (f) in the IVC-Sham-H and IVC-HDN group rats and (g) in the PV-Sham-H and PV-HDN group rats (n = 5 rats per group). The results are presented as the means ± SDs, n.s.: not significant, Ref.: reference. *$P < 0.05$ based on the two-tailed Student's *t*-test in (a), (d), and (e), the paired *t*-test in (b) and (c), and the two-tailed Student's *t*-test with Bonferroni correction in (f) and (g).

Supplementary Figure S11.

Figure 6a. p-eNOS^{Thr495}

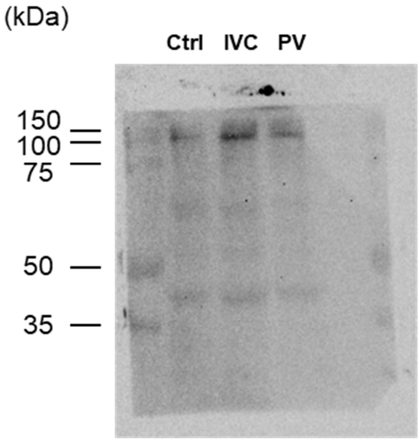

Figure 6a. eNOS

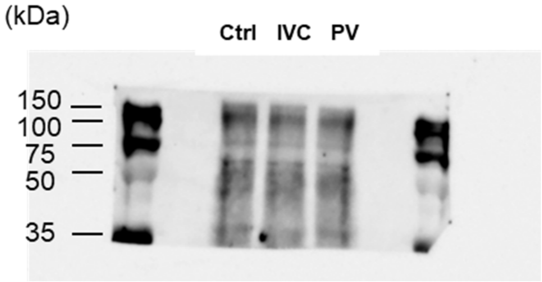

Supplementary Figure S11. The original uncropped immunoblots of phosphorylated eNOS (Thr495) and total eNOS shown in Fig. 6a.

Supplementary Figure S12

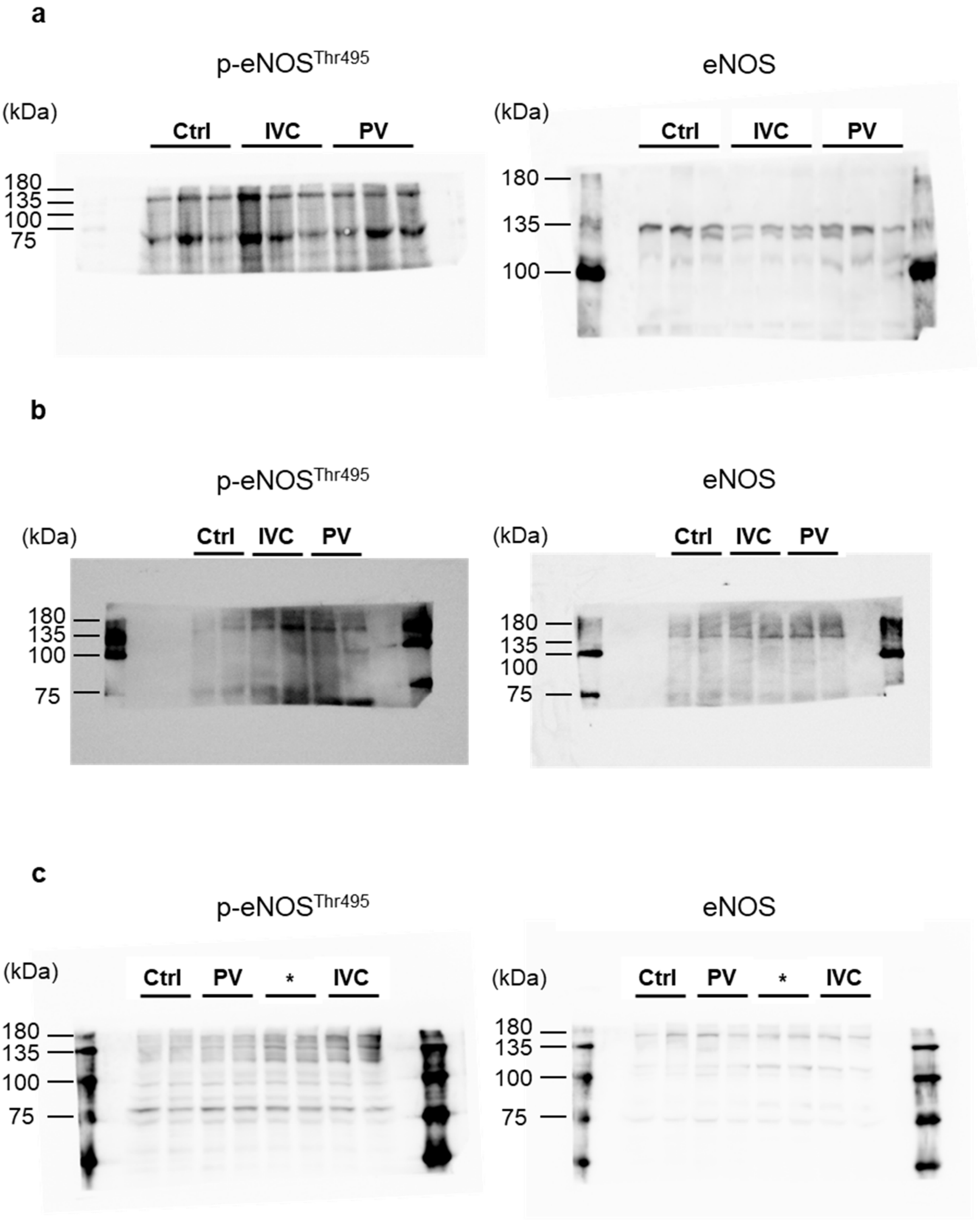

Supplementary Figure S12. All of the immunoblots of phosphorylated eNOS (Thr495) and eNOS used for quantification in Fig. 6b.

The (*) sample in (c) was excluded as it was irrelevant to the experimental results.

Supplementary Table S1.
Intervention details for each group

Group Name	Pre -treatment	Phosphate Intervention	Administration Route	Figure
Duo	none	Phosphate (80 µmol) + ^{32}P	duodenum	Figure 1a, 1d
Ctrl	none	Normal saline	portal vein	Figure 2a, 4a, S1
IVC	none	Phosphate (20 µmol)	inferior vena cava	Figure 2a, 4a
PV	none	Phosphate (20 µmol)	portal vein	Figure 2a, 4a, S1
Ctrl-BNX	BNX	Normal saline	portal vein	Figure 2e, 6c
IVC-BNX	BNX	Phosphate (20 µmol)	inferior vena cava	Figure 2e
PV-BNX	BNX	Phosphate (20 µmol)	portal vein	Figure 2e
IVC-^{32}P	None	Phosphate (20 µmol) + ^{32}P	inferior vena cava	Figure 3a, S7a
PV-^{32}P	None	Phosphate (20 µmol) + ^{32}P	portal vein	Figure 3a, S7a
Ctrl-PH	PH	Phosphate (20 µmol)	portal vein	Figure 3c
PV-PH	PH	Phosphate (20 µmol)	portal vein	Figure 3c
Ctrl-$GdCl_3$	$GdCl_3$	Normal saline	portal vein	Figure 3f
Ctrl-MCT	MCT	Normal saline	portal vein	Figure 3f
IVC-$GdCl_3$	$GdCl_3$	Phosphate (20 µmol)	inferior vena cava	Figure 3f
IVC-MCT	MCT	Phosphate (20 µmol)	inferior vena cava	Figure 3f
PV-$GdCl_3$	$GdCl_3$	Phosphate (20 µmol)	portal vein	Figure 3f
PV-MCT	MCT	Phosphate (20 µmol)	portal vein	Figure 3f
Ctrl-veh	vehicle	Normal saline	portal vein	Figure 5a
PV-veh	vehicle	Phosphate (20 µmol)	portal vein	Figure 3f, 5a
IVC-veh	vehicle	Phosphate (20 µmol)	inferior vena cava	Figure 5a
IVC-SCH	SCH	Phosphate (20 µmol)	inferior vena cava	Figure 5a
PV-SCH	SCH	Phosphate (20 µmol)	portal vein	Figure 5a
IVC-^{32}P-BNX	BNX	Phosphate (20 µmol) + ^{32}P	inferior vena cava	Figure 6c
PV-^{32}P-BNX	BNX	Phosphate (20 µmol) + ^{32}P	portal vein	Figure 6c
IVC-Sham-R	sham-RDN	Phosphate (20 µmol)	inferior vena cava	Figure 7a, S9
IVC-RDN	RDN	Phosphate (20 µmol)	inferior vena cava	Figure 7a, S9
PV-Sham-R	sham-RDN	Phosphate (20 µmol)	portal vein	Figure 7a, S9
PV-RDN	RDN	Phosphate (20 µmol)	portal vein	Figure 7a, S9
IVC-Sham-H	sham-HDN	Phosphate (20 µmol)	inferior vena cava	Figure 8a, S10
IVC-HDN	HDN	Phosphate (20 µmol)	inferior vena cava	Figure 8a, S10
PV-Sham-H	sham-HDN	Phosphate (20 µmol)	portal vein	Figure 8a, S10
PV-HDN	HDN	Phosphate (20 µmol)	portal vein	Figure 8a, S10
PV-100	none	Phosphate (100 µmol)	portal vein	Figure S1

PV-500	none	Phosphate (500 µmol)	portal vein	Figure S1
Ctrl-TPTX	TPTX	Normal saline	portal vein	Figure S4c
IVC-TPTX	TPTX	Phosphate (20 µmol)	inferior vena cava	Figure S4c
PV-TPTX	TPTX	Phosphate (20 µmol)	portal vein	Figure S4c

Intervention details for each group are summarized.

Abbreviations:

$GdCl_3$, gadolinium chloride; MCT, monocrotaline; vehicle, normal saline; SCH, SCH23390; BNX, bilateral nephrectomy; TPTX, thyroparathyroidectomy; PH, partial hepatectomy; sham-PH, sham operation of partial hepatectomy; RDN, renal denervation; sham-RDN, sham operation of renal denervation; HDN, hepatic denervation; sham-HDN, sham operation of hepatic denervation

Phosphate interventions:

Normal saline: NaCl solution (154 mM) (1.0 ml/animal).

Phosphate (80 µmol) + ^{32}P: Na_2HPO_4/NaH_2PO_4 solution (phosphate 80 mM, pH 7.4) containing radiolabeled phosphate (3700 Bq/ml) (1.0 ml/animal).

Phosphate (20 µmol) + ^{32}P: Na_2HPO_4/NaH_2PO_4 solution (phosphate 20 mM, pH 7.4) containing radiolabeled phosphate (3700 Bq/ml) (1.0 ml/animal).

Phosphate (20 µmol): Na_2HPO_4/NaH_2PO_4 solution (phosphate 20 mM, pH 7.4) (1.0 ml/animal).

Phosphate (100 µmol): Na_2HPO_4/NaH_2PO_4 solution (phosphate 100 mM, pH 7.4) (1.0 ml/animal).

Phosphate (500 µmol): Na_2HPO_4/NaH_2PO_4 solution (phosphate 500 mM, pH 7.4) (1.0 ml/animal).
